# Fungicide application increased copper-bioavailability and impaired nitrogen fixation through reduced root nodule formation on alfalfa

**DOI:** 10.1007/s10646-019-02047-9

**Published:** 2019-05-28

**Authors:** Martin Schneider, Katharina M. Keiblinger, Melanie Paumann, Gerhard Soja, Axel Mentler, Alireza Golestani-Fard, Anika Retzmann, Thomas Prohaska, Sophie Zechmeister-Boltenstern, Walter Wenzel, Franz Zehetner

**Affiliations:** 1 Institute of Soil Research, University of Natural Resources and Life Sciences, Peter-Jordan-Straße 82, 1190 Vienna, Austria; 2 Austrian Institute of Technology, Center for Energy, Konrad Lorenz-Straße 24, 3430 Tulln an der Donau, Austria; 3 Division of Agronomy, University of Natural Resources and Life Sciences, Konrad Lorenz-Straße 24, 3430 Tulln an der Donau, Austria; 4 Division of Analytical Chemistry, University of Natural Resources and Life Sciences, Konrad Lorenz-Straße 24, 3430 Tulln an der Donau, Austria; 5 Chair of General and Analytical Chemistry, Montanuniversität Leoben, Franz-Josef-Straße 18, 8700 Leoben, Austria; 6 Present Address: Institut für Bodenforschung, Schwackhöfer Haus, Peter-Jordan-Straße 82, 1190 Wien, Austria

**Keywords:** Endosymbiosis, Heavy metal, LA-ICP-MS, Pollutant, Toxicology, Trace element

## Abstract

Copper-based fungicides have been used for a long time in viticulture and have accumulated in many vineyard soils. In this study, incrementing Cu(OH)_2_-based fungicide application from 0.05 to 5 g Cu kg^−1^ on two agricultural soils (an acidic sandy loam (L, pH 4.95) and an alkaline silt loam (D, pH 7.45)) resulted in 5 times more mobile Cu in the acidic soil. The most sensitive parameters of alfalfa *(Medicago sativa*) growing in these soils were the root nodule number, decreasing to 34% and 15% of the control at 0.1 g Cu kg^−1^ in soil L and at 1.5 g Cu kg^−1^ in soil D, respectively, as well as the nodule biomass, decreasing to 25% and 27% at 0.5 g Cu kg^−1^ in soil L and at 1.5 g Cu kg^−1^ in soil D, respectively. However, the enzymatic N_2_-fixation was not directly affected by Cu in spite of the presence of Cu in the meristem and the zone of effective N_2_-fixation, as illustrated by chemical imaging. The strongly different responses observed in the two tested soils reflect the higher buffering capacity of the alkaline silt loam and showed that Cu mitigation and remediation strategies should especially target vineyards with acidic, sandy soils.

## Introduction

Salts of Cu are used in agriculture since more than 120 years. Either CuSO_4_ or more recently also Cu(OH)_2_ show superior fungicidal properties and a lack of resistances of fungi, compared to synthetic fungicides (Kühne et al. 2009). Their preventive applications mitigate losses in photosynthetically active leaf area in vineyards, mainly caused by the infection with downy mildew (*Plasmopara viticola*). Current maximum application rates of 3 kg Cu ha^−1^ on conventionally grown grapevine in Austria, or 6 kg Cu ha^−1^ according to EU-legislation (EC 2008) are not supposed to cause remarkable increases in soil Cu contents, compared to inputs through sewage sludge or manure. The majority of contaminations on agricultural soil was caused by application rates of 30 and even up to 80 kg Cu ha^−1^ before 1970 (Kühne et al. 2009). Nevertheless, soils may show Cu accumulation due to low harvest withdrawals of 3–30 mg kg^−1^ plant dry weight (dw) (Amelung et al. 2018) and because of long-term use of Cu-based fungicides, which is nowadays, especially in organic farming systems, still inevitable.

Especially former vineyards report total soil Cu concentrations of about 1030 mg kg^−1^ in France (Bravin et al. 2010; Michaud et al. 2007), 888 mg kg^−1^ in Austria (Berger et al. 2012), 493 mg kg^−1^ in Brazil (Girotto et al. 2014), 249 mg kg^−1^ in Australia (Pietrzak and McPhail 2004) and 200 mg kg^−1^ in Serbia (Ninkov et al. 2014). However, the probable no-effect levels calculated for soils range only from 55 to 155 mg total Cu kg^−1^ in Austria (Berger et al. 2012), whereas the German precautionary values only range from 20 to 60 mg total Cu kg^−1^ in sand or clay, respectively (BBodSchV 1999).

Besides the soil organic matter (SOM) content, representing the major sorption pool for Cu, the sorption of Cu is governed by soil pH, with stronger adsorption at pH > 4.5 (Lair et al. 2007; Lair et al. 2006) and higher mobility at pH < 4.5 (Amelung et al. 2018; Cornu et al. 2007). Therefore, the main proportions of Cu are retained in the uppermost soil layer in association with SOM and thus do not cause detrimental effects on deep rooting perennial crops. However, Cu toxicity can be observed for annual intercrops, or when replacing the perennial crops by annual ones (Michaud et al. 2007).

Cu is a redox-active essential core element in various enzymes and cofactors. It is necessary for the electron transfer in photosynthesis as well as for the final reduction of O_2_ in the respiration chain via cytochrome-cbb_3_-oxidase (Brennicke and Schopfer 2010; White 2012). Toxicity mainly occurs by proliferation of cell membranes and the subsequent catalytic production of reactive oxygen species (ROS) via the Fenton- /Haber-Weiss-reaction (Díaz et al. 2001; Halliwell and Gutteridge 1984; Mostofa and Fujita 2013).

Due to restrictions in the use of synthetic fungicides and mineral fertilizers, organic viticulture depends on Cu as well as on co-cropping with legumes. Since *Fabaceae* are Cu-sensitive (Dahlin et al. 1997), there is a trade-off between effective plant protection and efficient N_2_-fixation.

*Medicago sativa* (lucerne or alfalfa) is a perennial plant and N_2_-fixing via endosymbiotic rhizobia, which support growth, nutrient uptake and resilience (Ashrafi et al. 2014). *Rhizobium meliloti* colonize fine roots and form root nodules. Every known rhizobium utilizes enzymes for the reduction of N_2_ into ammonia, i.e. mainly the Mo/Fe-nitrogenase. Some strains are also capable to use V or only Fe as cofactors, if the availability of Mo is insufficient (Bellenger et al. 2014; Brennicke and Schopfer 2010; White 2012). To prevent the nitrogenases from inactivation trough free O_2_, the root nodules of legumes contain leghemoglobin to maintain microaerob conditions (Bellenger et al. 2014; Brennicke and Schopfer 2010).

It is known that Cu elevates N_2_-fixation up to 10 mg Cu kg^−1^ soil dw (Snowball et al. 1980). However, Baijukya and Semu (1998) showed decreasing biomass of *Phaseolus vulgaris* and plant-related number and biomass of nodules at application rates of 2.2 mg Cu kg^−1^ soil dw. The potential N_2_-fixation of alfalfa in perlite with nutrient solution at pH 6 was reduced by 97% at 10 mg Cu L^−1^ as CuSO_4_, with visual symptoms of plant toxicity at 100 mg L^−1^ (Porter and Sheridan 1981). Ippolito et al. (2011) observed no alfalfa growth at 500 mg Cu kg^−1^ (CuSO_4_) applied after 5 weeks of growth. In a CuSO_4_-ammended natural forest soil with pH 5.9, Caetano et al. (2016) obtained effective concentrations with 50% decrease in harvest at 93 to 291 mg Cu kg^−1^ soil dw for *Lactuca sativa* and for *Avena sativa*, respectively. The assumption of root proliferation (Voigt et al. 2006) being the main mechanism of Cu-toxicity is validated by (Chen et al. 2013) with Cu-concentrations presenting the first significant increase in the same CuSO_4_-treatments as the decreases in root elongation.

In spite of numerous studies about Cu affecting plant growth, data about effects of Cu(OH)_2_ on N_2_-fixation is still scarce. Therefore, the objective of this study was to identify thresholds for significant impacts of Cu(OH)_2_-application on different plant compartments and N_2_-fixation of alfalfa, a typical cover crop in vineyards. This was tested after application on a wide range of soil Cu concentrations in two soils with contrasting properties. We hypothesized that the bioavailability of added copper is higher in the acidic soil, therefore resulting in lower toxicity thresholds compared to the calcareous soil. In particular, it was further hypothesized, that increasing Cu levels beyond the thresholds result in (a) decreased biomass production of different plant compartments (shoot, root, nodule), (b) elevated plant tissue Cu concentrations and (c) depressed nitrogenase activity.

## Materials and methods

### Soil properties and experimental design

We used an acidic sandy loam from Lasberg (L), Upper Austria, with a pH of 4.95 (1:12.5, w/v, soil/0.01 M CaCl_2_) as well as an alkaline silt loam from Deutsch Jahrndorf (D), Burgenland, with a pH of 7.45 (Table 1).

**Table 1:** Basic properties of the studied soils

Soil	pH^a^	Clay	Sand	Silt	OC	N_t_^b^	P^c^	K^c^	Fe^d^	Mn^d^	Cu^e^
		----------/ % ------------			--/ g kg^−1^--		---------------/ mg kg^−1^--------------------				
L	4.95	7.8	66.4	25.8	16.4	1.66	87	129	279	64	5.2
D	7.45	16.8	28.6	54.6	17.6	1.79	122	402	185	413	18.6

^a^In 0.01 M CaCl_2_

^b^Total nitrogen

^c^In CAL—calcium acetate lactate

^d^In EDTA—ethylenediaminetetraacetic acid

^e^In AR—aqua regia

Topsoils were sampled in March 2015 and sieved to ≤1 cm. The unfertilized soils were filled into 4.8 L polypropylene (PP) pots (Ø 20 cm) at a bulk density of 1.2 g cm^−3^. The pots were equipped with a polyethylene fleece at the bottom, to prevent particle movement while flushing, and with 6 mm glass fiber wicks for passive watering. The wicks reached up to 50% of the pots height and down to the bottom of a black 5 L PP bucket, installed beneath the pots (Supp. Fig. 1). The buckets were filled with artificial rain water, i.e. desalinated water with 3 mg Ca L^−1^ (50% CaCl_2_ and 50% CaSO_4_). The greenhouse experiment was placed in a completely randomized design (Supp. Tab. 1) at temperatures from 7.7 to 37.2 °C (mean: 20.4 °C) and a relative humidity of 16.9 to 83.1% (mean: 57.4%).

Since the presence of nitrate may decrease root nodulation and might have accumulated through excess mineralization during pot preparation (Broos et al. 2004), nitrate was leached with water by flushing the pots two times (one week in between) with an amount equivalent to 200% of the maximum water holding capacity (WHC).

Two weeks after leaching, a commonly used fungicide (Funguran Progress®, Spiess-Urania, Hamburg, Germany), containing 53.7% Cu(OH)_2_, was applied as a suspension in water onto the topsoil (Supp. Fig. 2) to reach 90% WHC in concentrations of 0, 0.05, 0.1, 0.2, 0.5, 1.5 and 5 g Cu kg^−1^ soil dw with five replicates per concentration. One week after spiking, topsoils were scarified and inoculated with various rhizobial strains (Feldsaaten Freudenberger, Krefeld, Germany). On the 8th of May, two weeks after application of the fungicide, ten seeds of *M. sativa* cultivar. Plato (Feldsaaten Freudenberger) were sown per pot after inoculating the seeds with rhizobia to ensure nodulation. The plant number was reduced when the first four plants reached a height of 15 to 20 cm.

### Soil analyses

Pots were sampled over the whole height on the 14th and on the 92nd DAS with a 1-cm stainless steel auger (Supp. Fig. 3) and sieved to ≤2 mm immediately for microbial analyses (Keiblinger et al. 2018). Table 1 shows the basic topsoil properties according to Austrian standards (OENorm-L1061-2 2002; OENorm-L1079 1999; OENorm-L1085 2009; OENorm-L1095 2002) prior to sampling them in the field. The soil pH (OENorm-L1083 2006) was measured with 2 g of air-dried soil in 25 mL 0.01 M CaCl_2_ using a pH meter (pH 537, WTW GmbH Weilheim, Germany). Briefly, the organic carbon and total nitrogen according to (Brandstätter et al. 2013) was determined using a TOC/TN analyser (TOC-V CPHE200V, Shimadzu Corporation, Kyoto, Japan). Plant available phosphorus and potassium (OENorm-L1087 2004) were extracted with calcium acetate lactate. Potassium was determined in an atomic absorption spectrometer (AAS, Perkin Elmer 2100, MA, US) and phosphorus was measured spectro-photometrically using the molybdate blue staining method (Schinner et al. 1996).

Air-dried soil samples were used for Cu analyses in ethylenediaminetetraacetic acid- (EDTA) and CaCl_2_-extracts for both samplings, while Cu in diffusive gradients in thin films (DGT) was performed only for the second sampling.

#### Copper analyses in soil extracts

Samples of 5 g air-dried soil were extracted with 50 mL 0.05 M Na-EDTA, shaken for 2 h and filtered (Munktell 14/N). Solutes were measured in flame-AAS (AAnalyst 400, Perkin Elmer) and correspond to the organically complexable amount of Cu (OENorm-L1089 2005).

Neutral salt Cu extracts were prepared with 2.5 g air-dried soil and 50 mL 0.01 M CaCl_2_, corresponding to the easily soluble amount of Cu (Houba et al. 2000). The soil was equilibrated in solution overnight and shaken on the following day for 3 h, prior to filtration and measurement with flame-AAS (PineAAcle 900T, Perkin Elmer).

#### Copper quantification with the diffusive gradients in thin films technique (DGT)

DGT is an infinite sink approach, interpreting the concentration C_DGT_ as the time-averaged concentration of Cu at the interface of soil solution and DGT device. Free Cu ions are supplied by desorption from the soil solid phase and by mass flow or diffusion as well as dissociation of labile complexes in soil solution and within the diffusive gel of the DGT piston (Degryse et al. 2009; Harper et al. 1998).

Following Zhang (2005), the DGT sampling pistons consisted of 25-mm plastic sockets covered by a 400-µm thin Chelex 100 (sodium form, Sigma Aldrich) resin gel disc, an 800-µm thick polyacrylamide diffusive gel disc, covered by a 150-µm polyether sulfone membrane with 0.45 µm mesh (Sartorius). A cap with a soil contact window of Ø 20 mm kept these layer in place.

Soil samples were wetted to 60% WHC and incubated air tight for 24 h at 20 °C. Afterwards, the water content was raised to 90% WHC and DGT were deployed for 24 h. The resin gels were eluted in 1 M HNO_3_ and analyzed by ICP-MS (ELAN DRL-e SIEX, Perkin Elmer) with ^115^In as internal standard and 103 to 104% element recovery for Cu.

The concentration *C*_DGT_ (nmol cm^−3^) was calculated by the mass of metal *M* (nmol) bound by the resin gel and the diffusion layer thickness Δ*g* (0.095 cm), related to the diffusion coefficient *D*_w_ of Cu in water (5.29 × 10^−6^ cm^2^ s^−1^) and the deployment time *t* (s) as well as the exposing area of the DGT device *A* (cm^2^).

1 $$C_{{\mathrm{DGT}}} = M \times \frac{{\Delta g}}{{D_{\mathrm{w}} \times t \times A}}$$

Whereas the mass of metal *M* was calculated from the concentration determined in the eluate (µg cm^−3^) and the volume of the eluate *V*_eluate_ as well as of the resin disc *V*_disc_ (cm^3^), with further correction for elution efficiency using a recovery factor *f*_recovery_ of 0.82.

2 $${\mathrm{M = }}\frac{{{\mathrm{c}}_{{\mathrm{eluate}}} \times (V_{{\mathrm{eluate}}} + V_{{\mathrm{disc}}})}}{{f_{{\mathrm{recovery}}}}}$$

### Plant analyses

Measurements of shoot height (cm), chlorophyll density (SPAD-502PLUS, Konica Minolta Optics Inc., Osaka, Japan) and counting of internodial distances, ramification and flowering were conducted between the 81^st^ and the 84^th^ day after sowing (DAS), while plants were harvested at the 101^st^ and 102^nd^ DAS.

Samples of shoots, roots and nodules were dried at 70 °C to constant weight and ground. Roots and nodules were washed carefully before drying.

An amount of 0.2 g oven dried roots, shoots or nodules was digested with 10 mL of 65% nitric- and 70% perchloric acid in a ratio of 6:1 (v/v), filtered (Whatman 589/2) and Cu was analyzed in a graphite furnace (HGA 900, Perkin Elmer) AAS (AAS, AAnalyst 400).

### Nitrogenase activity

The potential nitrogenase activity was assessed using acetylene reduction assays (ARA) by sampling ten nodules of each pot at plant harvest. According to Quilliam et al. (2013) and Zechmeister-Boltenstern (1993), 20-mL headspace vials were filled with 2 cm^3^ washed and calcined fine granular sea sand and 1.55 mL of isotonic Ringer solution, containing, 2.25 mM Ca^2+^,155.7 mM Cl^−^, 4 mM K^+^ and 147.2 mM Na^+^ for preventing an osmotic shock, as well as 4 g glucose L^−1^ to avert substrate limitations (Supp. Fig. 4–5).

Acetylene replaced 10% of the headspace volume and after 24 h incubation at 20 °C, gas samples were analyzed in a gas chromatograph (Hewlett Packard 5890 Series II with Hewlett Packard 7694 Headspace Sampler, Wilmington, NC, USA) equipped with an Alumnia column (Agilent, Santa Clara, CA, USA) and a flame ionization detector (Hewlett Packard 6890). An external standard with 10 ppm ethylene was used. The amount of produced ethylene was related to the nodule number and the nodule volume, measured with a microscope (Olympus SZX, 10UC 30 camera, 72 dpi, Olympus Corporation, Tokyo, Japan).

### Root nodule imaging

A second experiment followed the toxicity experiment with a mixed soil sample of all five replicates from the previously contaminated silt loam (0.5 g Cu kg^−1^). The soil was filled into rhizotrons (Supp. Fig. 6) at 1.4 g cm^−3^ dry density and wetted to 100% WHC once. Rhizotrons were inclined to 10° and equipped with 6-mm glass fiber wicks for passive watering. Six seedlings of *M. sativa* were pre-germinated for three days before transferring into the rhizotrons. Root nodules were harvested after ten weeks of growth and washed in Millipore water with 20 s ultrasound treatment at 10% energy-output on continuous mode (HD2200 with 200 W, Bandelin electronics, Berlin, Germany). Nodules were frozen onto the cutting socket of a cryostat (CM3050, Leica, Bensheim, Germany) with liquid nitrogen and cut into 50-µm longitudinal and radial sections The nodule sections were air dried onto object slides and measured out with a microscope (Olympus SZX, 10UC 30).

The spatial distribution of ^13^C, ^63^Cu, ^57^Fe, ^97^Mo, ^55^Mn, ^32^S, and ^51^V within the sections was sampled by laser ablation (LA), using a neodymium doped yttrium aluminum garnet laser (NWR 193, New Wave Research, Fremont, CA, US) with a laser energy of 3.6 mJ cm^−2^ connected to an inductively coupled plasma - mass spectrometer (ICP-MS, ELEMENT XR, Thermo Scientific, Bremen, Germany) with Ar and He as carrier gasses. Root nodule images were generated by ablating parallel lines from the nodule surface. The resolution of 9.95 × 21.14 µm for the longitudinal section and 9.95 × 25.49 µm for the radial section was determined by the spot size of 10 µm, the scan speed of 5 µm s^−1^ and the dwell time of 1.99 s. Therefore, the counts of each data point of the scanned line consisted of 40 laser shots.

### Data evaluation

Data processing and LA-ICP-MS image generation was performed in R (R-Development-Core-Team 2015). The Tukey’s HSD (Honestly Significant Difference) test was used for identifying differences at p < 0.05 between treatments and for determining the concentration with the first significant response, i.e. the lowest observed adverse effect level (LOAEL). The Welch test was used for differences between sampling times. Freundlich coefficients and the effective concentration for harvest losses of 50% (EC_50_) were calculated by regressions performed with mean values, with the root mean square error (RMSE) describing the goodness of fit. The RMSE was normalized on the value range into the normalized root mean square error (NRMSE) for comparing models with different variables.

3 $${\mathrm{y = K}}_{\mathrm{F}} \times {\mathrm{x}}^{\frac{1}{{\mathrm{n}}}}$$

The Freundlich equation estimates the resupply of Cu in *x* (mg L^−1^) from a solid or adsorbed pool, i.e. from soil and labile complexes *y* (mg kg^−1^). The parameter n shapes the isotherm and *K*_F_ (L kg^−1^) is a constant for the adsorbed amount at a mobile fraction of 1 mg kg^−1^ (Freundlich 1909). The isotherms in our study are not identical to isotherms of adsorption experiments as we used C_DGT_-Cu instead of the solution concentration and EDTA-extractable Cu as the adsorbed fraction.

## Results

### Availability of copper by diffusive gradients in thin films and soil extracts

**Fig. 1:**
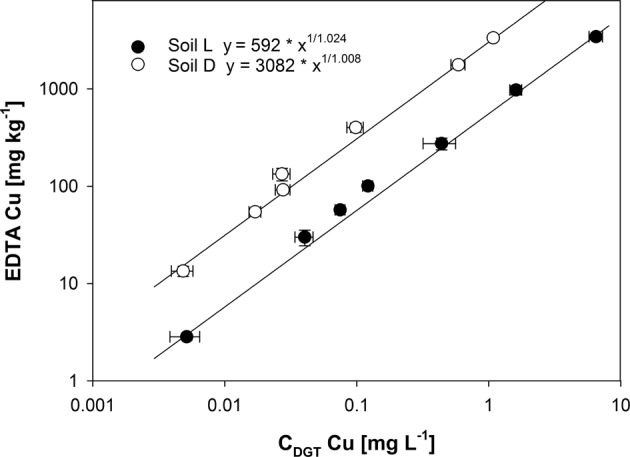
Relationship of 0.05 M EDTA-extractable Cu and Cu in diffusive gradients in thin films on the 92^nd^ day after sowing, according to Freundlich isotherms. The R^2^ was above 0.999 with *p* < 0.001 for both soils. The error bars represent standard errors (*n* = 5). The normalized root mean square error was 2.8% for soil L and 1.6% for soil D

Both soils showed strong increases in C_DGT_-Cu, CaCl_2_-extractable Cu and EDTA-extractable Cu with increasing fungicide application (Supp. Tab. 2). The EDTA-Cu and C_DGT_-Cu concentrations were consistently increasing along application rates with the first significant increase at 1.5 g kg^−1^. The ratio of the Freundlich coefficients K_F_ (Fig. 1, Supp. Fig. 7) indicated 5.09 to 5.34 (mean: 5.21) more bioavailable Cu present in the acidic soil. In another way, ratios (data not shown) of EDTA-Cu and C_DGT_-Cu indicated 4.6 to 5.6 (mean: 5.1) times more diffusive resupply and hence, higher plant-availability in soil L.

The EDTA- and CaCl_2_-extractable Cu did not differ between soils, whereas the CaCl_2_-Cu showed a more dynamic pattern as it increased steadily with application rates only in soil L at the first sampling. At the second sampling, the 0.05 and 0.1 g kg^−1^ treatments showed marginally lower concentrations than the control. The same was the case for the alkaline soil D for both samplings in the 0.05, 0.1 and 0.2 g kg^−1^ treatments.

### Soil pH

The soil pH (Table 2) of the first sampling consistently increased with Cu application, while the control showed slightly higher pH than the lower three treatments of soil L and the lower four treatments of soil D. However, the first significant increase was at 1.5 g kg^−1^ in soil L and at 5 g kg^−1^ in soil D, while the pH correlated positively with soil and plant Cu concentrations (*p* < 0.05; Supp. Tab. 3). Comparing between the two samplings, the pH in soil L increased in all treatments, but most strongly in the treatments with plant growth and in the highest treatment. The decline of protons in the 0.05–0.5 g kg^−1^ treatments of soil L was 2-fold to 7-fold compared with the highest two treatments, revealing that plants alkalinized the soil. Nevertheless, the difference between the sampling times was not significant at 1.5 g kg^−1^. In soil D, a slight acidification took place in every treatment, except at 1.5 g kg^−1^, where plant growth was strongly diminished and the pH significantly increased from the first to the second sampling.

**Table 2:** Soil pH (0.01 M CaCl_2_) at the first and second sampling on the 14^th^ and 92^nd^ day after sowing, respectively, and the decrease in proton concentration from the first to the second sampling

soil	Cu spiked g kg^−1^	1. pH ± SEM	2. pH ± SEM	[H^+^] decrease nM ± SEM
L	0.00	4.82 ± 0.03c	5.04 ± 0.19b	2.74 ± 4.20
	0.05	4.75 ± 0.01c	5.40 ± 0.10ab	13.5 ± 1.55**
	0.10	4.79 ± 0.01c	5.52 ± 0.12ab	12.6 ± 0.88*
	0.20	4.81 ± 0.02c	5.30 ± 0.08b	10.1 ± 1.41**
	0.50	4.85 ± 0.03c	5.48 ± 0.09ab	10.6 ± 1.21***
	1.50	5.07 ± 0.04b	5.52 ± 0.11ab	5.12 ± 1.16
	5.00	5.54 ± 0.07a	5.90 ± 0.07a	1.72 ± 0.57*
D	0.00	7.46 ± 0.09bc	7.10 ± 0.04c	−0.043 ± 0.009*
	0.05	7.29 ± 0.01c	7.22 ± 0.02bc	−0.010 ± 0.003
	0.10	7.30 ± 0.01c	7.21 ± 0.02bc	−0.011 ± 0.002
	0.20	7.34 ± 0.01c	7.19 ± 0.02bc	−0.018 ± 0.003*
	0.50	7.43 ± 0.01bc	7.32 ± 0.02b	−0.010 ± 0.003
	1.50	7.59 ± 0.03ab	7.76 ± 0.02a	0.008 ± 0.002**
	5.00	7.68 ± 0.02a	7.64 ± 0.05a	−0.002 ± 0.002

Different lower case letters following standard errors (SEM, *n* = 5) indicate significant differences between treatments within each soil and asterisks indicate significant differences between the two sampling times

*Significant with *p* < 0.05

**Significant with *p* < 0.01

***Significant with *p* < 0.001

### Biomass production of *Medicago sativa*

**Fig. 2:**
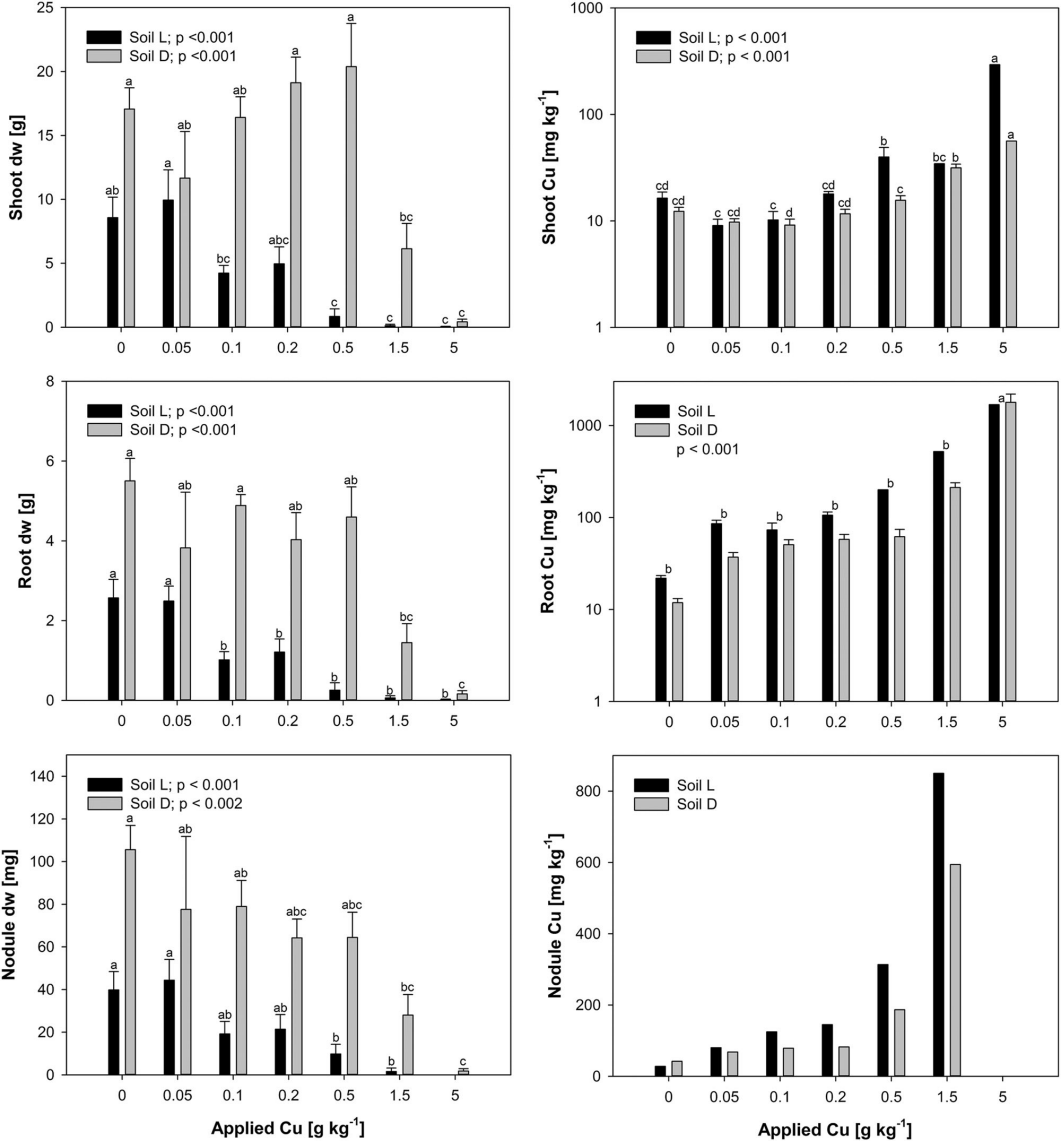
Biomass dry weights (dw) of shoot (top left), root (middle left) and root nodules (bottom left) per pot and Cu concentrations of shoot (top right), root (middle right) and root nodules (bottom right) after 101/102 days after sowing. Root Cu concentrations did not differ between soils. Root nodule Cu concentrations are single values of pooled samples within each treatment. Different letters above standard errors (*n* = 5) indicate significant differences between treatments within each soil, except for root Cu concentrations as soils were not significantly different

At 101 to 102 DAS, biomass production was generally higher in the alkaline soil (Fig. 2). But the biomass was in contrast to soil Cu and pH data more variable along the treatments and not constantly decreasing. The root and shoot biomass of soil L increased slightly in the 0.05 g kg^−1^ treatment to further decline. In soil D, however, the biomass production decreased in the first treatment to increase further up to the 0.5 g kg^−1^ treatment. The LOAEL of shoot and root biomass was 0.5 and 0.1 g Cu kg^−1^ soil in soil L and 1.5 g kg^−1^ in soil D, with shoot biomass decreasing to approximately 10% and 36%, respectively, and root biomass to 40% and 26%, respectively, relative to the control. But, related to the highest yield, the shoot biomass in soil L decreased already in the 0.1 g kg^−1^ treatment.

Fitting root and shoot biomass (Supp. Fig. 8) to extractable Cu presented C_DGT_-Cu being superior in soil D, but CaCl_2_-Cu fitted better in soil L. Regressions with EDTA-Cu showed the smallest error (i.e. 10 to 20%) for root nodule biomass in both soils.

The LOAEL for root nodule biomass was at 0.5 g Cu kg^−1^ in soil L and at 1.5 g Cu kg^−1^ in soil D, with biomass decreasing to 25% and 27%, respectively, relative to the control. For the nodule number (Supp. Tab. 4), the LOAEL was at 0.1 g Cu kg^−1^ in soil L and at 1.5 g Cu kg^−1^ in soil D, with decreases to 34% and 15%, respectively. In both soils, the nodule number and biomass were positively correlated with shoot and root biomass and with the growth parameters and negatively with CaCl_2_-Cu at the first sampling, while correlating negatively with all soil Cu analyses in soil D. Interestingly, both nodule parameters decreased more strongly than the below- or above ground biomass production.

The growth parameters (Supp. Fig. 9) were different between the soils, but seemed to be less affected by Cu. Shoot height, internodial distances and ramification showed the LOAEL at 0.5 g kg^−1^ in soil L and at 5 g kg^−1^ in soil D. Also the flowering was affected in this treatment in soil D. The chlorophyll density was responsive at 1.5 g kg^−1^ in soil L and at 5 g kg^−1^ in soil D. All these parameters were positively correlated with each other and with plant biomass in both soils (*p* < 0.05). Also all Cu measurements in both soils, except C_DGT_-Cu in soil L correlated with the growth factors. The shoot height and SPAD values of soil L were correlated with CaCl_2_-Cu at the first sampling only.

### Accumulation and translocation of copper in *Medicago sativa*

The shoot and root Cu concentrations (Supp. Fig. 10) of the 1.5 and 5 g kg^−1^ treatments needed to be analyzed as pooled samples of the whole treatment, since plant growth strongly diminished. In both soils, we observed slightly decreasing shoot Cu compared to the control up to 0.1 g kg^−1^ in soil L and until 0.2 g kg^−1^ in soil D, but not significantly. Shoot Cu increased at 0.5 g kg^−1^ in soil L and at 1.5 g kg^−1^ in soil D. The root Cu concentrations were continuously increasing with application rate and higher in the acidic soil L, but they were statistically similar in both soils. Both, shoot and root Cu concentrations were correlated with soil Cu, while CaCl_2_-Cu at the second sampling showed the highest correlation coefficient in soil D. In soil L, shoot Cu was stronger correlated with C_DGT_-Cu and root Cu with EDTA-Cu at the second sampling. In regressions of both soils (Supp. Tab. 5), C_DGT_-Cu presented the smallest error (NRMSE: 4.3%) for shoot Cu and the CaCl_2_-Cu concentrations at the second sampling seemed to result in non-linear regressions superior for root and nodule Cu.

The ratio of Cu in shoots and roots (Supp. Tab. 4) was similar in both soils and highest in the control, resulting in ratios of 0.77 in soil L and 1.08 in soil D, with mostly higher ratios in soil D. The Cu withdrawal (Supp. Tab 6) by below- and above ground biomass was overall higher in soil D, except for roots at 0.05 g kg^−1^. Taking both aspects together presented a stronger translocation of Cu into the shoots of plants from soil D, which increased up to 0.5 g kg^−1^ as the biomass.

Assuming the CaCl_2_-Cu to be the relevant fraction for plant uptake, the root uptake based on 4.8 kg soil dw accounted for 102% and 18% in the 0.05 and 0.1 g kg^−1^ treatment of soil L, respectively, of the decrease in CaCl_2_-Cu (−31 µg kg^−1^ in both treatments) compared to the control (Supp. Tab. 6). Since the shoot withdrawal decreased in response to Cu exposure, the whole plant uptake only accounted for 68% in the 0.05 g kg^−1^ treatment. In the alkaline soil, higher plant growth increased Cu withdrawals, while root uptake in the 0.05, 0.1 and 0.2 g kg^−1^ treatments accounted for 33, 80 and 98%, respectively, of the decreases in CaCl_2_-Cu (−48, −48 and −34 µg kg^−1^), shoot uptake for 6% in the 0.2 g kg^−1^ treatment, and the whole plant uptake for 74 and 105% in the 0.1 and 0.2 g kg^−1^ treatment, respectively. On the other hand, the increases of CaCl_2_-Cu in the following two treatments (+117 and +444 µg kg^−1^) were compensated through root uptake by 32% and 10% and through plant uptake by nearly 50% and 9%, respectively. In the acidic soil, root compensation was less than 5% of the increase in CaCl_2_-Cu.

The contribution of root nodules to the changes in CaCl_2_-Cu were neglectable, caused by their low weight, as the uptake of roots and shoots exceeded nodule uptake by mean 42–45 fold in soil L and 30–34 fold in soil D. The nodule Cu concentrations of pooled samples per treatment increased according to Cu application and were correlated with soil Cu and root Cu. Interestingly, the nodules obtained in average 42% higher Cu concentrations than roots from soil L and 137% in soil D, respectively.

### Nitrogen fixation and element distribution in root nodules

**Fig. 3:**
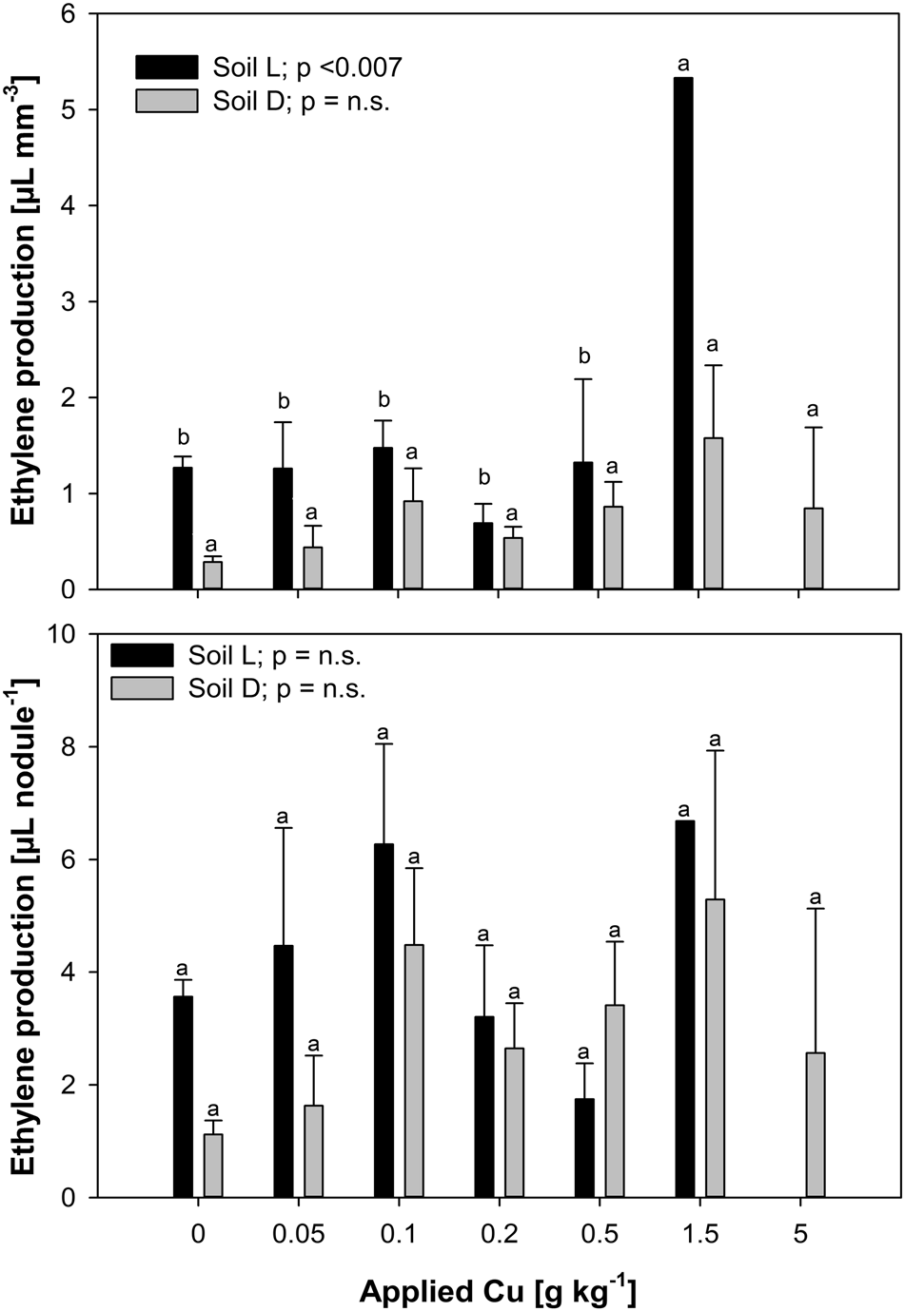
Ethylene production through acetylene reduction by root nodules related to nodule size (top) and nodule number (bottom) 101/102 days after sowing. Different letters above standard errors (*n* = 5, see Supp. Tab. 4) indicate significant differences between treatments within each soil. Ethylene production related to the nodule number was not significantly different between soils or treatments

The ARA reached from 0 to 5.33 (mean: 1.05) µL ethylene mm^−3^ nodule^−1^ (Fig. 3) and was different between soils, whereas increased ethylene production was observed in the acidic soil L, compared with the alkaline soil D. However, if the highest ethylene production result in the 1.5 g Cu kg^−1^ treatment of soil L was excluded, because only one measurement of a cluster-like nodule was available, the ethylene production was not correlated with soil Cu. Whereas inlusion of this measurement caused a positive correlation with nodule Cu in both soils and with soil Cu and root Cu in soil L.

Related to the nodule number, the ethylene production ranged from 0 to 12.8 (mean: 3.43) µL ethylene nodule^−1^ and was neither different between soils, nor between treatments and showed no systematic correlations. These results under consideration of the morphology of the nodules from the 1.5 g Cu kg^−1^ treatment in soil L justified the exclusion of the highest ethylene production result and the conclusion of no relation of the ARA with soil Cu.

**Fig. 4:**
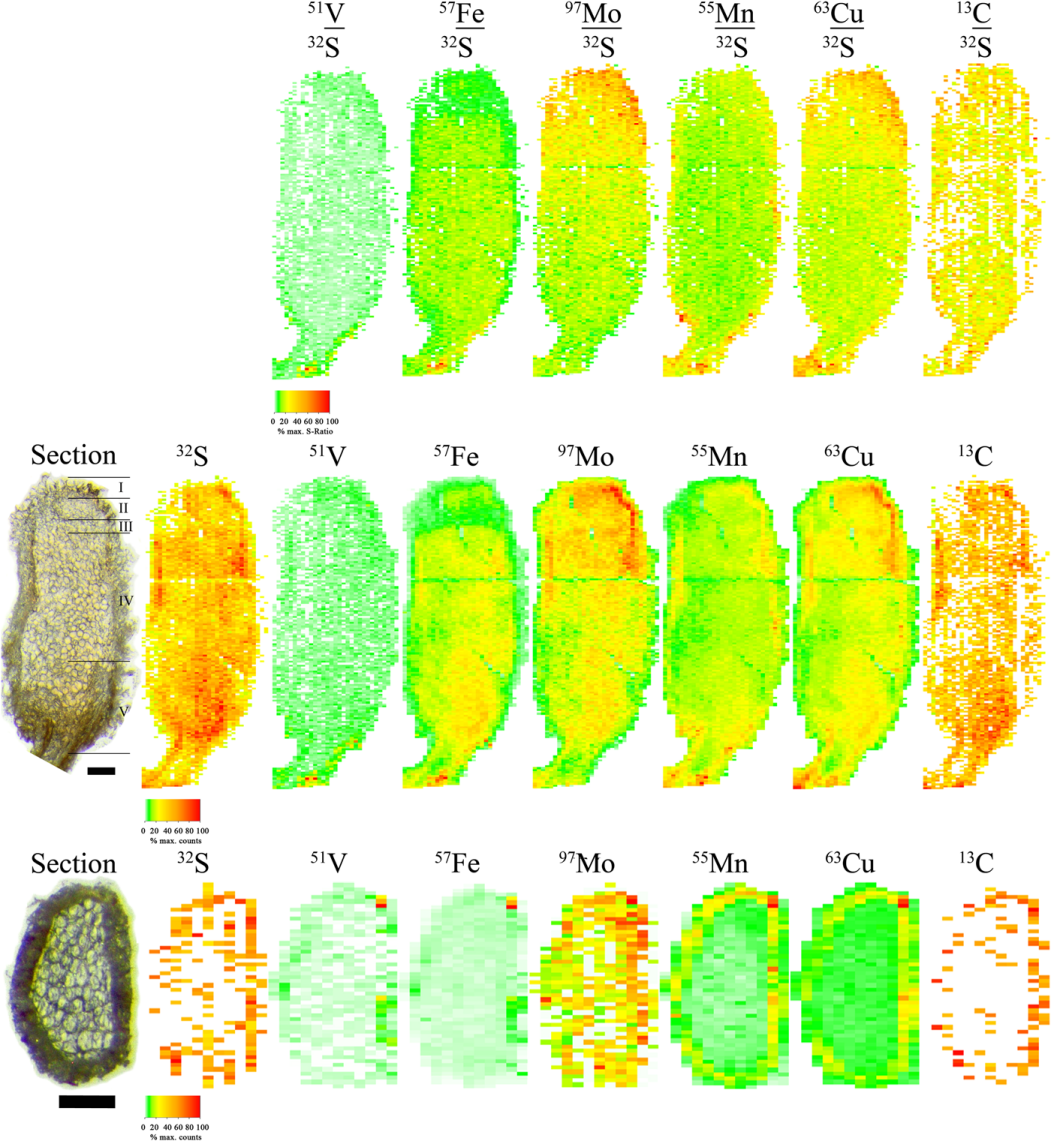
Images of elemental distributions in root nodules of *Medicago sativa* as measured by LA-ICP-MS with abundances based on ^32^S distribution (top), absolute counts of elements in the longitudinal section (middle) and absolute counts in the radial section (bottom). Results are scaled on the maximum measured counts or ^32^S-based ratio of each image for reasons of comparability. Labels in the micrograph indicate the cortex (I), meristem (II), infection zone (III), N_2_-fixing zone (IV) and the senescing zone (V) followed by the root. The empty line in the longitudinal section resulted from a test run. The radial section was taken from another nodule and shows the cortex and cells. Blank cells correspond to values below the detection limit. The black bar corresponds to 160 µm

Chemical imaging of the radial nodule section (Fig. 4) yielded ^32^S mostly below detection limit. However, the absence of ^32^S or ^13^C was not caused by a lack of matrix, as absolute counts of ^97^Mo showed a fairly consistent distribution across the nodule. We mainly found ^63^Cu and ^55^Mn in the cortex, whereas ^57^Fe and ^51^V occurred in very low amounts.

The longitudinal section of the nodule also contained ^55^Mn mostly in the cortex. The meristem contained most of the ^63^Cu and ^97^Mo, related to ^32^S, while ^57^Fe, e.g. in nitrogenase, was homogenously distributed from the post-meristem, i.e. the infection zone, towards the root, while ^97^Mo declined. Small amounts of ^51^V were found in the meristem and the ^32^S-poor zone, which also contains leghemoglobin (Brennicke and Schopfer 2010). In the latter, the highest abundances of ^57^Fe, ^51^V, ^63^Cu and ^55^Mn were found. Comparable results were achieved by single linescans from longitudinal section of other nodules (Supp. Fig. 11–12).

Absolute counts of the longitudinal section gave the same information, whereas higher intensities of ^63^Cu and ^97^Mo were found in the zone of N_2_-fixation, which contained the highest density of ^32^S and ^57^Fe.

## Discussion

### The behavior of copper in soil

The relation of EDTA-Cu to C_DGT_-Cu showed more bioavailable Cu present in the acidic soil (Fig. 1). This indicates lower sorption capacity (Lair et al. 2006) for the fungicide in the acidic sandy soil L and stronger adsorption in the finer textured alkaline soil D. The pH, clay and silt contents of soil D could explain its stronger Cu sorption (Elbana and Selim 2011; Kabata-Pendias 2004; Lair et al. 2007; Lair et al. 2006; Lindsay 1979). In acidic soils, less pH-dependent binding sites are available for adsorption onto SOM, which governs Cu mobility to a large extend. Especially in alkaline environments, the precipitation as Cu–carbonates, -oxides and -hydroxides as well as cupric ferrite strongly reduces [Cu^2+^] activity (Lindsay 1979), while also the sorption onto mineral surfaces, e.g. Fe-, Al-, Mn-oxides and –hydroxides decreases Cu availability (Amelung et al. 2018; Lair et al. 2007; Lair et al. 2006; Wenzel et al. 2011).

### Plant responses affecting copper availability

Plants prevent metal uptake from soil by immobilization through organic ligands or by influencing the pH-dependent mobility by releasing protons or anions. The latter causes surface deprotonation of soil particles with variable charges, which decreases Cu mobility and therefore toxicity (Bravin et al. 2009; Cornu et al. 2007; Wenzel et al. 2011; White 2012).

The pH increased in the current study between the two samplings in the acidic soil (Table 2) indicating these rhizosphere processes. Wherease the increase in pH was observed for the whole pots and not only in the rhizosphere, where pH effects might have been much more pronounced, at least in the acidic soil (Michaud et al. 2007). Bravin et al. (2009) described for durum wheat increasing pH from 4.6 to 7.4 in a vineyard soil with 184 mg total Cu kg^−1^, reaching more than 6 mm into the rhizosphere. Increases of soil pH by 0.09–0.27 units were observed by Cornu et al. (2007) with tomato on contaminated acidic vineyard soils with pH 4.3 and 43–47 mg Cu kg^−1^ in EDTA. On calcareous soil (pH 8.1–8.7 and 51–176 mg Cu kg^−1^), they observed pH decreases by 0.08–0.1 units, while Tao et al. (2004) mentioned that a change by less than 0.5 units at a soil pH of 8.12 and 126 mg total Cu kg^−1^ does not affect Cu fractionation. However, decreasing the proton concentration in soil solution decreases [Cu^2+^] activity and triggers sequestration and precipation as Cu minerals (Lindsay 1979) triggered by plants.

In the alkaline soil D, the pH also increased with increasing Cu application, but was mostly lower at the second sampling indicating a slight acidification during plant growth. As the pH contrary increased with time at 1.5 g Cu kg^−1^, but not at the highest application rate, it can be concluded that plants facing Cu toxicity utilize rhizosphere alkalization even in alkaline environments.

### Root copper sorption capacity, translocation and physiological consequences

Plant growth was more sensitive in soil L, which revealed higher activity of Cu in acidic environments, whereas the growth factors representing stunted growth as indication of Cu toxicity (Díaz et al. 2001; Zorn et al. 2013) were less sensitive than the diminishing plant biomass.

The LOAEL for shoot biomass, i.e. 0.5 and 0.1 g Cu kg^−1^ soil in soil L and 1.5 g kg^−1^ in soil D, was identical with the increase of shoot Cu in both soils, indicating root membrane damage and subsequent higher symplastic uptake (Cestone et al. 2012) followed by oxidative stress (Díaz et al. 2001; Halliwell and Gutteridge 1984). A comparable pattern was observed by Chen et al. (2013) for the relative root elongation of grapevine exposed to 1–25 µM [Cu^2+^]. The order of sensitivity according to EC_50_, i.e. the effective concentration for harvest losses of 50%, (Supp. Fig. 8) followed nodule > root > shoot biomass in soil D and the opposite in soil L. The corresponding EC_50_-concentrations are listed in Supp. Tab. 7. and in general, they exceeded the EC_50_ of Caetano et al. (2016).

In this context, the use of artificial rainwater might have influenced the uptake and overestimated the observed toxicity thresholds, since (i) Ca^2+^ is competing with Cu for sorption sites at the roots, causing less Cu uptake and toxicity alleviation (Degryse et al. 2009; Wenzel et al. 2011; Wu and Hendershot 2010) and (ii) besides HPO_4_^2-^, SO_4_^2-^ and Cl^-^ are preferentially taken up by metal-exposed alfalfa plants for complexing free Cu ions (Peralta-Videa et al. 2002). Also, the fact that roots in compacted, potted soil grew preferentially at the pot-sided soil surfaces, might have lowered the observed thresholds.

Our data suggest the CaCl_2_-Cu concentrations to be directly affected by plants throught root uptake and translocation into above ground biomass(Supp. Tab. 6). Physiological consequences of root Cu sorption were similar in both soils, indicated by the ratios of shoot to root biomass and tissue Cu concentrations (Supp. Tab. 4). However, the capacity of the plant for Cu uptake without facing toxicity, represented by the plant withdrawal (Supp. Tab. 6), was higher in the alkaline soil D, except at 0.05 g kg^−1^. Interestingly, more Cu was retained in roots under acidic growth condition, while plants from the alkaline soil translocated Cu to a larger extend into the shoot. Also the slight decrease in shoot Cu with increasing root Cu in response to application rates of up to 0.1 g kg^−1^ in soil L and up to 0.2 g kg^−1^ soil D indicates effective attenuation of Cu translocation into aerial parts (Michaud et al. 2007).

Adsorption to the root by influencing variable charges remains small compared to the absorbed amount (Degryse et al. 2012). However, root cell wall binding of up to 1 g kg^−1^ (Bravin et al. 2010) mainly controls Cu mobility in plants growing in contaminated media (Cestone et al. 2012; Michaud et al. 2007; Wenzel et al. 2011).

Especially in calcareous soil, root adsorption might be higher due to less proton competition for surface charges, with subsequent lower toxicity thresholds (Degryse et al. 2009; Michaud et al. 2007; Peralta-Videa et al. 2002). Tissue concentrations of Cu (Supp. Fig. 10) in this study were in the range of durum wheat (Bravin et al. 2010). Related to CaCl_2_-Cu, tissue Cu in the alkaline soil was higher than in the acidic soil, as observed by Michaud et al. (2007). At the LOAEL for biomass yields, root and nodule Cu concentrations were higher in the alkaline soil, while shoot Cu was lower than in the acidic soil. However, related on treatments or EDTA-extractable Cu, all tissues from plants growing in the acidic soil contained more Cu, as observed by Cornu et al. (2007).

### Copper influencing the nitrogen fixation

The N_2_-fixation by root nodules was strongly diminished indirectly through their declining biomass and the root nodule number. As a consequence of impaired root development they appeared as the most responsive parameters to Cu in our study. They were remarkably reduced at C_DGT_-Cu higher than 1.6 mg L^−1^ in this study, which is lower than the 10 mg Cu L^−1^ solution concentration resulting in 97% inhibition of ARA, reported by Porter and Sheridan (1981).

However, ethylene production was not related to any soil extractable Cu fraction. The potential nitrogenase activity measured via ARA in this study (Fig. 3) was higher in the acidic soil. This is (i) pH dependent, as Porter and Sheridan (1981) reported highest values for ARA at pH 3 and (ii) caused by higher Fe availability for N_2_-fixation (Bellenger et al. 2014; Brennicke and Schopfer 2010; White 2012).

Broos et al. (2004), who used ^15^N with clover, did observe metal toxicity on N_2_-fixation in a sewage sludge amended soil, whereas no differentiation could be made between direct detrimental effects of Cu, Zn, Cd and excess of mineral-N or co-contaminants. In metal salt amended soil, they showed depressed N_2_-fixation, if the host plant was not able to survive. Comparable results were obtained by Chaudri et al. (2008) for the population size of *R. leguminosarum* with sludge cake and metal salt spiked sewage sludge. Dahlin et al. (1997) showed decreases in auto- and heterotrophic N_2_-fixation in a long-term experiment free of crops with soils treated with metal contaminated sewage sludge and metal salts. Commonly, these authors reported Cu with the highest relative increase, compared to other metals, with negative correlation to the potential N_2_-fixation. However, no distinction between pH-effect, soil N-increase and specific heavy metal response could be made. Contrary, population sizes of *R. leguminosarum* were not correlated with Cu and slightly increasing with higher metal salt addition (Dahlin et al. 1997).

In our study, the root nodules showed higher Cu uptake than roots, except for the 0.05 g kg^−1^ treatment in soil L, without affecting nitrogenase activity. Higher internalization into nodules was also shown by Snowball et al. (1980) for up to 10 mg Cu kg^−1^ in soil, and is caused by high-affinity metal chelating proteins, making nodules an important metal sink (González-Guerrero et al. 2016).

Our nodule images by LA-ICP-MS (Fig. 4) were in good agreement to Rodríguez-Haas et al. (2013). They also showed sporadic Fe, and therefore nitrogenase in the infection zone and declining Fe concentrations in the senescence zone, with no Fe in the meristem and the epidermal layer.

High abundances of ^63^Cu and ^97^Mo in the meristem might be related to their involvement as cofactors in a number of enzymatic processes. These are condensed in regions of high metabolic activity (Arora et al. 2010; Hille et al. 1998; Preisig et al. 1996). The declining ^97^Mo/^32^S ratios towards the root might indicate the preferential use of Mo/Fe-nitrogenase by younger symbiosomes, while older ones tend to use the alternative Fe/Fe- or V/Fe-nitrogenase.

Likely due to high contents of leghemoglobin, the region close to the root seemed to buffer high levels of ^63^Cu, ^55^Mn, ^57^Fe and ^51^V compared to the whole nodule. Despite the possibility of inducing ROS, ^63^Cu was present in the zone of N_2_-fixation. Therein, rhizobia metabolize NO_3_^−^, which is further reduced by the Fe- or Mo-containing NO_3_^−^-reductase to NO_2_^−^ (Arora et al. 2010; Hille et al. 1998). Since NO_2_^−^ is toxic to the nitrogenase, it must be reduced by NO_2_^−^-reductase, which contains Cu as core element. The supply of Cu could therefore elevate nitrogenase activity, which is supported by increasing activity of NO_3_^−^- and NO_2_^−^-reductase, uptake hydrogenase and acetylene reduction by a free living *Sinorhizobium meliloti* in yeast extract mannitol agar containing 6.35 g L^−1^ Cu (Arora et al. 2010). These findings and considerations by chemical imaging illustrate the strong involvement of Cu during N_2_-fixation.

## Summary and conclusions

This work reported plant responses in soil after application of a Cu-based fungicide at moderate to very high concentrations, with the highest treatment being more strongly contaminated than former vineyards (Bravin et al. 2010; Michaud et al. 2007). We found more mobile Cu in an acidic sandy loam compared to an alkaline fine-textured silt loam. Depending on soil properties, notably pH and texture, the toxicity thresholds for alfalfa growth were much lower in the acidic soil and were in line with increases in plant tissue Cu concentrations. Our results also revealed the involvement of plant physiological responses affecting the mobility of Cu in these contaminated soils.

While this study could not achieve a positive or negative evaluation regarding effect of Cu on the enzymatic N_2_-fixation, it was thoroughly shown, that the nodule number and biomass was reduced, most likely as a consequence of impaired root development, at 0.1 g Cu kg^−1^ in the acidic soil and at 1.5 g kg^−1^ in the alkaline soil. The formation of root nodules consequently represented the most sensitive parameter to Cu in this work.

With respect to a sustainable soil use, the observed responses emphasize a careful and advised soil Cu management, especially in the more sensitive acidic, sandy soils, on which mitigation and remediation strategies should to focus. Assuming the current annual Cu application limits of 6 kg Cu ha^−1^ (EC 2008) and expecting plant withdrawals of 2–3 kg ha^−1^ the mentioned toxicity thresholds would be reached after 38–51 years in the acidic soil and after 573–764 years in the alkaline soil.

## Supplementary information

Supplementary information
