# Supplementary material for: Fungicide application increased copper-bioavailability and impaired nitrogen fixation through reduced root nodule formation on alfalfa
Source: Ecotoxicology. 2019 May 28;28(6):599–611. doi: 10.1007/s10646-019-02047-9 (PMC6647429; doi:10.1007/s10646-019-02047-9)
Supplement: Supplementary file 1 — Supplementary information [file 10646_2019_2047_MOESM1_ESM.docx]

# Fungicide application increased copper-bioavailability and impaired nitrogen fixation through reduced root nodule formation on alfalfa

# Ecotoxicology

Martin Schneider^1,2,3*^; Katharina M. Keiblinger^1^; Melanie Paumann^1^; Gerhard Soja^2^ ; Axel Mentler^1^ ; Alireza Golestani-Fard^1,3^; Anika Retzmann^4^; Thomas Prohaska^5^; Sophie Zechmeister-Boltenstern^1^; Walter Wenzel^1^; Franz Zehetner^1^

^1^ Institute of Soil Research, University of Natural Resources and Life Sciences, Peter-Jordan-Straße 82, 1190 Vienna, Austria

^2^ Austrian Institute of Technology, Department for Energy, Konrad Lorenz-Straße 24, 3430 Tulln an der Donau, Austria

^3^ Division of Agronomy, University of Natural Resources and Life Sciences, Konrad Lorenz-Straße 24, 3430 Tulln an der Donau, Austria

^4^ Division of Analytical Chemistry, University of Natural Resources and Life Sciences, Konrad Lorenz-Straße 24, 3430 Tulln an der Donau, Austria

^5^ Chair of General and Analytical Chemistry, Montanuniversität Leoben, Franz-Josef-Strasse 18, 8700 Leoben, Austria

*corresponding author. Phone: +43 1 47654-95121. E-mail address: martin.schneider@boku.ac.at.

# Supplementary material

| **Supp. Tab. 1** The experimental setup. | | | | | | | | | | | | | | | | | | | |
| --- | --- | --- | --- | --- | --- | --- | --- | --- | --- | --- | --- | --- | --- | --- | --- | --- | --- | --- | --- |
| **^1^0.1 e** | **F^4^c^5^** | **0a** | **5c** | *Fc* | **0.2a** | *0.05e* | **Fd** | *5a* | *1.5d* | **0.05d** | **0b** | **0.5a** | *0.5a* | *0.05d* | *5e* | **1.5b** | *0e* | **5e** | *Fe* |
| *^2^0.2e* | **0.1a** | *0b* | **1.5e** | *0a* | *1.5a* | *0.1d* | **Fb** | *0c* | *0.2a* | **0.2d** | *Fd* | **5a** | *0.5d* | *0.05c* | **Fe** | *0.1c* | **0.2e** | **0.5c** | **0.05a** |
| *0.5***^3^***b* | *Fa* | *0.05a* | **0.5d** | **0.5b** | *1.5c* | *0.5c* | **0.1d** | *5b* | **0.5e** | *0.05b* | **0.1b** | **1.5c** | **0.05b** | **0.2c** | *0.5e* | *0.2c* | **1.5d** | **0.1c** | *5d* |
| **1.5a** | **0.05c** | **0.2b** | *0.2b* | *0.1e* | **0d** | *1.5e* | **Fa** | *0.1a* | *5c* | **0.05e** | *Fb* | *0.2d* | *0d* | **5b** | **0e** | *1.5b* | *0.1b* | **5d** | **0c** |
| ^1^ bold - soil from Deutsch Jahrndorf.  ^2^ italics - soil from Lasberg.  ^3^ 0 to 5 - concentration of applied Cu (g kg^-1^).  ^4^ F – 0.5 g kg^-1^ inoculated with arbuscular mycorrhiza fungi (not studied within this work).  ^5^ a to e - replicates. | | | | | | | | | | | | | | | | | | | |

| **Supp. Tab. 2** Means of Cu concentrations measured by diffusive gradients in thin films (C_DGT_), 92^nd^ days after sowing, and extracted with 0.05 M EDTA and 0.01 M CaCl_2_. The 1. and 2. sampling was on the 14^th^ and on the 92^nd^ day after sowing, respectively. Different lower case letters following standard errors (SEM, n=5) indicate significant differences between treatments within each soil and asterisks indicate significant differences between the two sampling times. | | | | | | | | | | | | | | | | | | | | | | | |  |
| --- | --- | --- | --- | --- | --- | --- | --- | --- | --- | --- | --- | --- | --- | --- | --- | --- | --- | --- | --- | --- | --- | --- | --- | --- |
| soil | Cu spiked | C_DGT_-Cu |  | SEM |  | 1. EDTA-Cu |  | SEM |  |  | 2. EDTA-Cu |  | SEM |  | 1. CaCl_2_-Cu |  | SEM |  |  | 2. CaCl_2_-Cu |  | SEM |  | |
|  | g kg^-1^ | µg L^-1^ |  |  |  | mg kg^-1^ |  |  | n.s. |  | mg kg^-1^ |  |  | n.s. | µg kg^-1^ |  |  |  |  | µg kg^-1^ |  |  |  | |
| L | 0.00 | 5.15 | ± | 1.31 | c | 1.86 | ± | 0.10 | c |  | 2.84 | ± | 0.22 | c | 85.0 | ± | 0.90 | c |  | 183 | ± | 2.41 | c | |
|  | 0.05 | 40.3 | ± | 6.26 | c | 39.6 | ± | 12.8 | c |  | 29.9 | ± | 5.46 | c | 409 | ± | 20.0 | c |  | 152 | ± | 8.01 | c | |
|  | 0.10 | 74.9 | ± | 6.50 | c | 82.0 | ± | 10.5 | c |  | 57.1 | ± | 3.63 | c | 1040 | ± | 25.5 | c | * | 152 | ± | 3.52 | c | |
|  | 0.20 | 121 | ± | 10.7 | c | 216 | ± | 33.5 | c | * | 100 | ± | 7.76 | c | 3110 | ± | 46.8 | c | * | 419 | ± | 5.77 | c | |
|  | 0.50 | 440 | ± | 122 | bc | 606 | ± | 117 | bc |  | 274 | ± | 37.9 | c | 34000 | ± | 1410 | b |  | 4910 | ± | 329 | c | |
|  | 1.50 | 1610 | ± | 166 | b | 2230 | ± | 413 | b | * | 973 | ± | 105 | b | 54200 | ± | 230 | ab | *** | 17600 | ± | 308 | b | |
|  | 5.00 | 6510 | ± | 751 | a | 7080 | ± | 1040 | a | * | 3440 | ± | 92.13 | a | 73500 | ± | 303 | a |  | 51800 | ± | 479 | a | |
|  |  |  |  |  |  |  |  |  |  |  |  |  |  |  |  |  |  |  |  |  |  |  |  | |
| D | 0.00 | 4.89 | ± | 0.91 | c | 7.52 | ± | 0.09 | c |  | 13.3 | ± | 1.60 | c | 178 | ± | 7.21 | c |  | 103 | ± | 2.65 | c | |
|  | 0.05 | 17.2 | ± | 1.76 | c | 48.4 | ± | 10.8 | c |  | 54.1 | ± | 3.08 | c | 40.4 | ± | 1.14 | c |  | 54.5 | ± | 1.68 | c | |
|  | 0.10 | 27.9 | ± | 3.56 | c | 77.5 | ± | 7.97 | c |  | 91.0 | ± | 7.82 | c | 44.1 | ± | 2.04 | c |  | 54.5 | ± | 2.42 | c | |
|  | 0.20 | 27.4 | ± | 4.13 | c | 171 | ± | 37.7 | c |  | 132 | ± | 19.7 | c | 86.2 | ± | 2.89 | c |  | 68.5 | ± | 1.79 | c | |
|  | 0.50 | 99.0 | ± | 14.0 | c | 632 | ± | 34.5 | bc | * | 397 | ± | 42.9 | c | 310 | ± | 3.19 | bc |  | 220 | ± | 4.35 | c | |
|  | 1.50 | 595 | ± | 72.1 | b | 1660 | ± | 216 | b |  | 1750 | ± | 123 | b | 731 | ± | 8.29 | b |  | 547 | ± | 10.3 | b | |
|  | 5.00 | 1090 | ± | 56.6 | a | 2740 | ± | 531 | a |  | 3320 | ± | 206 | a | 1390 | ± | 25.0 | a |  | 1230 | ± | 13.0 | a | |
| n.s. - no significant difference between soils.  * - significant with p < 0.05.  *** - significant with p < 0.001. | | | | | | | | | | | | | | | | | | | | | | | |  |

| **Supp. Tab. 3** Pearson’s product-moment correlation coefficients (r) for the measured parameters in both soils, within soil L and within soil D including the highest ethylene production value of soil L. Bold numbers indicate significant correlations with p-values below 0.001. Bold italic numbers indicate significant correlations with p-values below 0.05. | | | | | | | | | | | | | | | | | | | | |
| --- | --- | --- | --- | --- | --- | --- | --- | --- | --- | --- | --- | --- | --- | --- | --- | --- | --- | --- | --- | --- |
| **Both** | 1.pH | 2.pH | height | nodulation | internodial distances | SPAD | ethylen µL mm-3 | ethylen µL nodule-1 | nodules | nodule dw | root dw | plant dw | nodule Cu | root Cu | plant Cu | 1.EDTA-Cu | 2.EDTA-Cu | 1.CaCl2-Cu | 2.CaCl2-Cu | C_DGT_ Cu |
| 2.pH | **0.988** | 1.000 |  |  |  |  |  |  |  |  |  |  |  |  |  |  |  |  |  |  |
| height | 0.351 | 0.294 | 1.000 |  |  |  |  |  |  |  |  |  |  |  |  |  |  |  |  |  |
| nodulation | 0.258 | 0.211 | **0.984** | 1.000 |  |  |  |  |  |  |  |  |  |  |  |  |  |  |  |  |
| internodial distances | 0.350 | 0.300 | **0.988** | **0.991** | 1.000 |  |  |  |  |  |  |  |  |  |  |  |  |  |  |  |
| SPAD | 0.248 | 0.220 | **0.882** | **0.926** | **0.924** | 1.000 |  |  |  |  |  |  |  |  |  |  |  |  |  |  |
| ethylen µL mm-3 | -0.396 | -0.367 | ***-0.626*** | ***-0.693*** | ***-0.721*** | **-0.831** | 1.000 |  |  |  |  |  |  |  |  |  |  |  |  |  |
| ethylen µL nodule-1 | -0.360 | -0.287 | -0.215 | -0.230 | -0.285 | -0.296 | ***0.697*** | 1.000 |  |  |  |  |  |  |  |  |  |  |  |  |
| nodules | 0.530 | 0.435 | **0.815** | ***0.753*** | **0.781** | ***0.594*** | -0.522 | -0.503 | 1.000 |  |  |  |  |  |  |  |  |  |  |  |
| nodule dw | ***0.539*** | 0.455 | **0.857** | **0.803** | **0.828** | ***0.644*** | -0.545 | -0.459 | **0.986** | 1.000 |  |  |  |  |  |  |  |  |  |  |
| root dw | ***0.550*** | 0.464 | **0.888** | **0.828** | **0.856** | ***0.647*** | -0.533 | -0.395 | **0.959** | **0.980** | 1.000 |  |  |  |  |  |  |  |  |  |
| plant dw | ***0.546*** | 0.473 | **0.901** | **0.836** | **0.871** | ***0.648*** | -0.517 | -0.324 | **0.883** | **0.906** | **0.963** | 1.000 |  |  |  |  |  |  |  |  |
| nodule Cu | -0.095 | -0.025 | ***-0.770*** | **-0.855** | **-0.807** | **-0.813** | **0.854** | ***0.562*** | ***-0.615*** | ***-0.619*** | ***-0.620*** | ***-0.577*** | 1.000 |  |  |  |  |  |  |  |
| root Cu | 0.082 | 0.114 | ***-0.749*** | ***-0.775*** | ***-0.753*** | ***-0.634*** | 0.160 | -0.022 | ***-0.552*** | ***-0.603*** | ***-0.592*** | ***-0.591*** | **0.951** | 1.000 |  |  |  |  |  |  |
| plant Cu | -0.129 | -0.116 | ***-0.610*** | ***-0.666*** | ***-0.648*** | ***-0.576*** | 0.330 | -0.021 | -0.422 | -0.461 | -0.455 | -0.453 | ***0.799*** | ***0.750*** | 1.000 |  |  |  |  |  |
| 1.EDTA-Cu | -0.028 | 0.003 | ***-0.731*** | **-0.798** | ***-0.771*** | ***-0.712*** | 0.533 | 0.274 | ***-0.540*** | ***-0.575*** | ***-0.561*** | ***-0.545*** | **0.989** | **0.863** | **0.948** | 1.000 |  |  |  |  |
| 2.EDTA-Cu | 0.204 | 0.255 | ***-0.700*** | ***-0.745*** | ***-0.703*** | ***-0.581*** | 0.170 | 0.086 | ***-0.562*** | ***-0.588*** | ***-0.574*** | ***-0.556*** | **0.841** | **0.949** | ***0.751*** | **0.890** | 1.000 |  |  |  |
| 1.CaCl2-Cu | -0.385 | -0.371 | **-0.798** | **-0.849** | **-0.847** | **-0.893** | **0.841** | 0.292 | -0.530 | ***-0.582*** | ***-0.594*** | ***-0.597*** | ***0.767*** | ***0.548*** | **0.784** | **0.794** | 0.511 | 1.000 |  |  |
| 2.CaCl2-Cu | -0.243 | -0.238 | ***-0.654*** | ***-0.725*** | ***-0.719*** | ***-0.718*** | **0.939** | 0.432 | -0.427 | -0.472 | -0.470 | -0.471 | ***0.822*** | ***0.659*** | **0.953** | **0.920** | ***0.644*** | **0.908** | 1.000 |  |
| C_DGT_ Cu | -0.148 | -0.132 | ***-0.668*** | ***-0.735*** | ***-0.721*** | ***-0.677*** | ***0.782*** | 0.384 | -0.462 | -0.503 | -0.498 | -0.496 | **0.960** | ***0.762*** | **0.985** | **0.970** | ***0.763*** | **0.846** | **0.982** | 1.000 |
|  |  |  |  |  |  |  |  |  |  |  |  |  |  |  |  |  |  |  |  |  |
| **L** | 1.pH | 2.pH | height | nodulation | internodial distances | SPAD | ethylen µL mm-3 | ethylen µL nodule-1 | nodules | nodule dw | root dw | plant dw | nodule Cu | root Cu | plant Cu | 1.EDTA-Cu | 2.EDTA-Cu | 1.CaCl2-Cu | 2.CaCl2-Cu | C_DGT_ Cu |
| 2.pH | ***0.784*** | 1.000 |  |  |  |  |  |  |  |  |  |  |  |  |  |  |  |  |  |  |
| height | ***-0.756*** | -0.733 | 1.000 |  |  |  |  |  |  |  |  |  |  |  |  |  |  |  |  |  |
| nodulation | ***-0.816*** | -0.727 | **0.989** | 1.000 |  |  |  |  |  |  |  |  |  |  |  |  |  |  |  |  |
| internodial distances | ***-0.814*** | -0.743 | **0.981** | **0.997** | 1.000 |  |  |  |  |  |  |  |  |  |  |  |  |  |  |  |
| SPAD | -0.715 | -0.640 | ***0.932*** | **0.958** | **0.972** | 1.000 |  |  |  |  |  |  |  |  |  |  |  |  |  |  |
| ethylen µL mm-3 | ***0.933*** | 0.419 | -0.708 | ***-0.803*** | ***-0.844*** | ***-0.891*** | 1.000 |  |  |  |  |  |  |  |  |  |  |  |  |  |
| ethylen µL nodule-1 | 0.413 | 0.398 | -0.090 | -0.178 | -0.247 | -0.289 | 0.651 | 1.000 |  |  |  |  |  |  |  |  |  |  |  |  |
| nodules | -0.621 | -0.747 | ***0.874*** | ***0.833*** | ***0.819*** | 0.716 | -0.500 | -0.191 | 1.000 |  |  |  |  |  |  |  |  |  |  |  |
| nodule dw | -0.702 | -0.740 | ***0.939*** | ***0.913*** | ***0.899*** | ***0.803*** | -0.609 | -0.202 | **0.976** | 1.000 |  |  |  |  |  |  |  |  |  |  |
| root dw | -0.633 | -0.754 | ***0.931*** | ***0.887*** | ***0.874*** | ***0.782*** | -0.545 | -0.145 | **0.982** | **0.989** | 1.000 |  |  |  |  |  |  |  |  |  |
| plant dw | -0.657 | -0.706 | ***0.949*** | ***0.912*** | ***0.895*** | ***0.803*** | -0.574 | -0.113 | **0.959** | **0.992** | **0.991** | 1.000 |  |  |  |  |  |  |  |  |
| nodule Cu | **0.962** | 0.541 | ***-0.891*** | ***-0.944*** | **-0.964** | **-0.972** | ***0.939*** | 0.439 | -0.719 | ***-0.799*** | ***-0.760*** | ***-0.777*** | 1.000 |  |  |  |  |  |  |  |
| root Cu | **0.990** | ***0.829*** | -0.718 | ***-0.772*** | ***-0.772*** | -0.667 | ***0.933*** | 0.426 | -0.595 | -0.659 | -0.603 | -0.613 | **0.997** | 1.000 |  |  |  |  |  |  |
| plant Cu | **0.952** | ***0.779*** | -0.616 | -0.662 | -0.653 | -0.519 | 0.496 | -0.267 | -0.502 | -0.564 | -0.511 | -0.525 | 0.712 | **0.976** | 1.000 |  |  |  |  |  |
| 1.EDTA-Cu | **0.995** | ***0.818*** | -0.727 | ***-0.784*** | ***-0.784*** | -0.682 | ***0.953*** | 0.445 | -0.599 | -0.668 | -0.608 | -0.622 | **0.996** | **0.999** | **0.971** | 1.000 |  |  |  |  |
| 2.EDTA-Cu | **0.992** | ***0.818*** | -0.710 | ***-0.766*** | ***-0.765*** | -0.658 | ***0.954*** | 0.453 | -0.585 | -0.653 | -0.593 | -0.606 | **0.997** | **1.000** | **0.978** | **0.999** | 1.000 |  |  |  |
| 1.CaCl2-Cu | ***0.902*** | ***0.783*** | **-0.952** | **-0.979** | **-0.979** | ***-0.930*** | ***0.826*** | 0.179 | ***-0.761*** | ***-0.849*** | ***-0.816*** | ***-0.840*** | ***0.948*** | ***0.875*** | ***0.786*** | ***0.883*** | ***0.869*** | 1.000 |  |  |
| 2.CaCl2-Cu | **0.996** | ***0.816*** | -0.743 | ***-0.801*** | ***-0.802*** | -0.705 | ***0.958*** | 0.434 | -0.602 | -0.677 | -0.616 | -0.633 | **0.992** | **0.998** | **0.964** | **0.999** | **0.998** | ***0.897*** | 1.000 |  |
| C_DGT_ Cu | **0.988** | ***0.813*** | -0.689 | -0.746 | -0.745 | -0.634 | ***0.958*** | 0.456 | -0.566 | -0.634 | -0.574 | -0.587 | **0.996** | **0.999** | **0.984** | **0.998** | **0.999** | ***0.855*** | **0.995** | 1.000 |
|  |  |  |  |  |  |  |  |  |  |  |  |  |  |  |  |  |  |  |  |  |
| **D** | 1.pH | 2.pH | height | nodulation | internodial distances | SPAD | ethylen µL mm-3 | ethylen µL nodule-1 | nodules | nodule dw | root dw | plant dw | nodule Cu | root Cu | plant Cu | 1.EDTA-Cu | 2.EDTA-Cu | 1.CaCl2-Cu | 2.CaCl2-Cu | C_DGT_ Cu |
| 2.pH | ***0.817*** | 1.000 |  |  |  |  |  |  |  |  |  |  |  |  |  |  |  |  |  |  |
| height | ***-0.813*** | ***-0.765*** | 1.000 |  |  |  |  |  |  |  |  |  |  |  |  |  |  |  |  |  |
| nodulation | ***-0.833*** | ***-0.787*** | **0.996** | 1.000 |  |  |  |  |  |  |  |  |  |  |  |  |  |  |  |  |
| internodial distances | ***-0.762*** | -0.745 | **0.996** | **0.991** | 1.000 |  |  |  |  |  |  |  |  |  |  |  |  |  |  |  |
| SPAD | -0.688 | -0.607 | ***0.915*** | ***0.894*** | ***0.907*** | 1.000 |  |  |  |  |  |  |  |  |  |  |  |  |  |  |
| ethylen µL mm-3 | 0.479 | ***0.830*** | -0.334 | -0.353 | -0.311 | -0.236 | 1.000 |  |  |  |  |  |  |  |  |  |  |  |  |  |
| ethylen µL nodule-1 | 0.176 | 0.582 | -0.054 | -0.068 | -0.036 | -0.051 | ***0.925*** | 1.000 |  |  |  |  |  |  |  |  |  |  |  |  |
| nodules | ***-0.759*** | ***-0.946*** | ***0.820*** | ***0.834*** | ***0.810*** | ***0.769*** | -0.736 | -0.525 | 1.000 |  |  |  |  |  |  |  |  |  |  |  |
| nodule dw | ***-0.769*** | ***-0.907*** | ***0.860*** | ***0.882*** | ***0.852*** | ***0.800*** | -0.631 | -0.417 | **0.979** | 1.000 |  |  |  |  |  |  |  |  |  |  |
| root dw | ***-0.796*** | ***-0.906*** | ***0.921*** | ***0.943*** | ***0.921*** | ***0.774*** | -0.561 | -0.295 | ***0.927*** | **0.962** | 1.000 |  |  |  |  |  |  |  |  |  |
| plant dw | ***-0.756*** | ***-0.805*** | ***0.946*** | ***0.948*** | ***0.950*** | ***0.757*** | -0.437 | -0.154 | ***0.778*** | ***0.806*** | ***0.923*** | 1.000 |  |  |  |  |  |  |  |  |
| nodule Cu | ***0.822*** | **0.992** | ***-0.818*** | ***-0.864*** | -0.714 | -0.378 | ***0.909*** | 0.732 | ***-0.900*** | ***-0.877*** | ***-0.896*** | -0.728 | 1.000 |  |  |  |  |  |  |  |
| root Cu | ***0.757*** | 0.597 | ***-0.940*** | ***-0.934*** | ***-0.926*** | **-0.965** | 0.158 | -0.057 | -0.727 | ***-0.800*** | ***-0.810*** | ***-0.797*** | **0.983** | 1.000 |  |  |  |  |  |  |
| plant Cu | ***0.919*** | ***0.824*** | **-0.959** | **-0.969** | ***-0.933*** | ***-0.892*** | 0.429 | 0.155 | ***-0.867*** | ***-0.906*** | ***-0.922*** | ***-0.874*** | **0.983** | ***0.934*** | 1.000 |  |  |  |  |  |
| 1.EDTA-Cu | ***0.917*** | ***0.898*** | ***-0.931*** | ***-0.944*** | ***-0.905*** | ***-0.860*** | 0.558 | 0.291 | ***-0.932*** | ***-0.947*** | ***-0.941*** | ***-0.860*** | **0.991** | ***0.879*** | **0.986** | 1.000 |  |  |  |  |
| 2.EDTA-Cu | ***0.905*** | ***0.863*** | **-0.960** | **-0.970** | ***-0.937*** | ***-0.885*** | 0.496 | 0.231 | ***-0.901*** | ***-0.932*** | ***-0.945*** | ***-0.893*** | **0.999** | ***0.916*** | **0.996** | **0.993** | 1.000 |  |  |  |
| 1.CaCl2-Cu | ***0.941*** | ***0.837*** | ***-0.943*** | **-0.952** | ***-0.912*** | ***-0.878*** | 0.455 | 0.176 | ***-0.870*** | ***-0.896*** | ***-0.905*** | ***-0.855*** | ***0.963*** | ***0.914*** | **0.997** | **0.989** | **0.991** | 1.000 |  |  |
| 2.CaCl2-Cu | ***0.906*** | ***0.804*** | **-0.963** | **-0.968** | ***-0.938*** | ***-0.916*** | 0.408 | 0.141 | ***-0.862*** | ***-0.898*** | ***-0.908*** | ***-0.865*** | **0.985** | ***0.948*** | **0.998** | **0.982** | **0.992** | **0.995** | 1.000 |  |
| C_DGT_ Cu | ***0.906*** | ***0.868*** | **-0.962** | **-0.972** | ***-0.939*** | ***-0.876*** | 0.503 | 0.236 | ***-0.898*** | ***-0.928*** | ***-0.949*** | ***-0.906*** | **0.996** | ***0.910*** | **0.994** | **0.991** | **0.999** | **0.989** | **0.989** | 1.000 |

| **Supp. Tab. 4** Means of shoot to root biomass and Cu concentration ratios and root nodule number. Different letters following standard errors (SEM, n=5) indicate significant differences between treatments within the same soil. | | | | | | | | | | | | | |
| --- | --- | --- | --- | --- | --- | --- | --- | --- | --- | --- | --- | --- | --- |
|  | | | | | | | | | | | | | |
| soil | Cu spiked  g kg^-1^ | shoot/  root | | SEM |  | shoot/  root Cu | | SEM |  | nodule number | | SEM |  |
|  |  |  |  |  | n.s. |  |  |  | n.s. |  |  |  |  |
| L | 0.00 | 3.36 | ± | 0.25 | a | 0.77 | ± | 0.12 | a | 169 | ± | 41.0 | a |
|  | 0.05 | 3.94 | ± | 0.66 | a | 0.11 | ± | 0.02 | b | 158 | ± | 34.8 | ab |
|  | 0.10 | 4.53 | ± | 0.49 | a | 0.14 | ± | 0.02 | b | 57.8 | ± | 18.9 | bc |
|  | 0.20 | 4.15 | ± | 0.18 | a | 0.15 | ± | 0.04 | b | 51.4 | ± | 11.9 | bc |
|  | 0.50 | 3.94 | ± | 0.54 | a | 0.20 | ± | 0.05 | b | 33.6 | ± | 26.6 | c |
|  | 1.50 | 2.38 | ±^3^ | 0.72 | a | 0.07 | * |  |  | 6.2 | * |  | c |
|  | 5.00 | 3.98 | ±^3^ | 2.72 | a | 0.17 | * |  |  |  |  |  |  |
|  | | | | | | | | | | | | | |
| D | 0.00 | 3.11 | ± | 0.12 | a | 1.08 | ± | 0.14 | a | 448 | ± | 37.8 | a |
|  | 0.05 | 3.61 | ± | 0.46 | a | 0.27 | ± | 0.02 | b | 311 | ± | 122 | ab |
|  | 0.10 | 3.43 | ± | 0.42 | a | 0.19 | ± | 0.03 | b | 303 | ± | 42.7 | ab |
|  | 0.20 | 5.36 | ± | 1.22 | a | 0.22 | ± | 0.03 | b | 304 | ± | 42.8 | ab |
|  | 0.50 | 4.44 | ± | 0.11 | a | 0.29 | ± | 0.06 | b | 215 | ± | 43.0 | abc |
|  | 1.50 | 4.29 | ±^4^ | 0.22 | a | 0.15 | ±^4^ | 0.01 | b | 69.0 | ± | 26.9 | bc |
|  | 5.00 | 2.79 | ± | 0.29 | a | 0.04 | ± | 0.02 | b | 7.4 | ± | 4.6 | c |
| n.s. - no significant difference between soils.  * - only one value available.  ^3^ - only three values available.  ^4^ - only four values available. | | | | | | | | | | | | | |

| **Supp. Tab. 5** Regression coefficients (y = a + bx) and quality parameters for the relation of tissue Cu concentrations with C_DGT_-Cu [µg L^-1^], 0.05 M EDTA-extractable Cu [mg kg^-1^] and 0.01 M CaCl_2_-extractable Cu [mg kg^-1^] at the 92^nd^ day after sowing. The p-values were lower than 0.01. | | | | | | | | | | | | |
| --- | --- | --- | --- | --- | --- | --- | --- | --- | --- | --- | --- | --- |
| tissue Cu | a |  | SEM^e^ | b |  | SEM | soil Cu | R^2^ | RMSE^f^ | NRMSE^g^ | |  |
|  |  |  |  |  |  |  |  |  |  | Both | L | D |
| shoot | 5.59 | ± | 16.3 | 0.05 | ± | 0.01 | EDTA-Cu | 0.528 | 47.4 | 16.7 | 17.7 | 94.1 |
| root |  |  |  | 0.47 | ± | 0.04 |  | 0.922 | 613 | 34.6 | 38.1 | 33.5 |
| nodules | 84.7 | ± | 49.5 | 0.41 | ± | 0.08 |  | 0.678 | 565 | 68.7 | 70.5 | 99.4 |
|  |  |  |  |  |  |  |  |  |  |  |  |  |
| shoot | 8.26 | ± | 3.92 | 0.04 | ± | 0.002 | C_DGT_-Cu | 0.968 | 12.4 | 4.3 | 6.1 | 4.9 |
| root |  |  |  | 0.30 | ± | 0.06 |  | 0.627 | 555 | 31.3 | 45.5 | 12.1 |
| nodules | 82.6 | ± | 24.7 | 0.52 | ± | 0.05 |  | 0.914 | 951 | 51.5 | 149 | 38 |
|  |  |  |  |  |  |  |  |  |  |  |  |  |
| shoot^h^ | 42.9 | ± | 6.12 | 0.40 | ± | 0.06 | CaCl_2_-Cu | 0.775 | 37.6 | 13.2 | 18.6 | 12.1 |
| root^h^ | 180 | ± | 42.7 | 0.56 | ± | 0.12 |  | 0.545 | 440 | 24.8 | 10.1 | 33.8 |
| nodules^h^ | 232 | ± | 31.5 | 0.45 | ± | 0.11 |  | 0.816 | 134 | 16.3 | 9.1 | 31.5 |
| ^e^ SEM - standard error of mean.  ^f^ RMSE - root mean square error.  ^g^ NRMSE -normalized root mean square error in %.  ^h^ y = ax^b^ | | | | | | | | | | | | |

| **Supp. Tab. 6** Comparison of plant uptake with changes in 0.01 M CaCl_2_-extractable Cu. The shoot withdrawal and the root uptake were calculated from the biomass and the Cu content based on the soil dry weight. Changes related to the control of CaCl_2_-extractable Cu, shoot withdrawal and root uptake are shown. The plant uptake is the sum of shoot withdrawal and root uptake. Different letters following standard errors (SEM, n=5) indicate significant differences between treatments within the same soil. | | | | | | | | | | | | | | | | |
| --- | --- | --- | --- | --- | --- | --- | --- | --- | --- | --- | --- | --- | --- | --- | --- | --- |
| soil | Cu spiked | shoot uptake | | |  | root uptake | | |  | CaCl_2_-Cu | shoot uptake | | root uptake | | plant uptake | |
|  | g kg^-1^ | µg kg^-1^ |  | SEM |  | µg kg^-1^ |  | SEM |  | change  µg kg^-1^ | change  µg kg^-1^ | % of CaCl_2_-Cu  change | change  µg kg^-1^ | % of CaCl_2_-Cu  change | change  µg kg^-1^ | % of CaCl_2_-Cu  change |
| L | 0.00 | 28.7 | ± | 6.10 | a | 11.1 | ± | 1.02 | b | 0 | 0 | 0 | 0 | 0 | 0 | 0 |
|  | 0.05 | 18.3 | ± | 4.03 | ab | 42.7 | ± | 4.52 | a | -30.8 | -10.4 | 33.7 | 31.57 | -103 | 21.2 | -68.8 |
|  | 0.10 | 9.60 | ± | 3.06 | bc | 16.8 | ± | 5.84 | b | -30.8 | -19.1 | 62.1 | 5.71 | -18.6 | -13.4 | 43.5 |
|  | 0.20 | 17.3 | ± | 5.11 | abc | 24.6 | ± | 5.85 | ab | 236 | -11.4 | -4.85 | 13.53 | 5.74 | 2.09 | 0.89 |
|  | 0.50 | 5.39 | ± | 3.24 | bc | 10.6 | ± | 7.79 | b | 4730 | -23.3 | -0.49 | -0.55 | -0.01 | -23.9 | -0.51 |
|  | 1.50 | 0.89 | ±^3^ | 0.76 | bc | 6.79 | ±^3^ | 5.79 | b | 17400 | -27.8 | -0.16 | -4.32 | -0.02 | -32.2 | -0.18 |
|  | 5.00 | 2.92 | ±^4^ | 0.99 | c | 6.80 | ±^3^ | 4.72 | b | 51600 | -25.8 | -0.05 | -4.31 | -0.01 | -30.1 | -0.06 |
|  |  |  |  |  |  |  |  |  |  |  |  |  |  |  |  |  |
| D | 0.00 | 44.7 | ± | 8.08 | ab | 13.6 | ± | 1.96 | a | 0 | 0 | 0 | 0 | 0 | 0 | 0 |
|  | 0.05 | 23.8 | ± | 8.05 | bc | 29.8 | ± | 13.9 | a | -48.3 | -21.0 | 43.4 | 16.2 | -33.5 | -4.79 | 9.92 |
|  | 0.10 | 31.9 | ± | 6.43 | abc | 52.6 | ± | 9.24 | a | -48.3 | -12.9 | 26.6 | 38.9 | -80.6 | 26.1 | -54.0 |
|  | 0.20 | 47.0 | ± | 7.03 | ab | 47.4 | ± | 7.54 | a | -34.3 | 2.26 | -6.58 | 33.8 | -98.5 | 36.0 | -105 |
|  | 0.50 | 65.0 | ± | 12.9 | a | 51.6 | ± | 4.79 | a | 117 | 20.3 | 17.4 | 38.0 | 32.4 | 58.3 | 49.8 |
|  | 1.50 | 37.8 | ±^4^ | 11.2 | abc | 58.6 | ±^4^ | 17.5 | a | 445 | -6.9 | -1.56 | 44.9 | 10.1 | 38.0 | 8.54 |
|  | 5.00 | 4.96 | ± | 2.43 | c | 35.1 | ± | 8.39 | a | 1130 | -39.8 | -3.53 | 21.4 | 1.90 | -18.3 | -1.63 |
| ^3^ - only three values available.  ^4^ - only four values available. | | | | | | | | | | | | | | | | |

| **Supp. Tab. 7:** Regression coefficients and quality parameters for describing percentage responses of plant growth to Cu (y = a + b * x^c^). As explaining variables C_DGT_-Cu [µg L^-1^] and 0.05 M EDTA-Cu [mg kg^-1^] and 0.01 M CaCl_2_- Cu [mg kg^-1^] were used. | | | | | | | | | | | | | | | |
| --- | --- | --- | --- | --- | --- | --- | --- | --- | --- | --- | --- | --- | --- | --- | --- |
|  | soil | y | a |  | SEM^a^ | b |  | SEM | c |  | SEM | adj. R^2^ | p | RMSE^b^ | EC_50_^c^ |
| C_DGT_-Cu | D | shoot | 85 | ± | 7.7 | -0.08 | ± | 0.02 |  |  |  | 0.784 | 0.005 | 14.2 | 445 |
|  |  | root | 85 | ± | 5.6 | -0.08 | ± | 0.01 |  |  |  | 0.880 | 0.001 | 10.4 | 435 |
|  |  | nodule | 76 | ± | 6.1 | -0.07 | ± | 0.01 |  |  |  | 0.833 | 0.003 | 11.3 | 363 |
|  |  | | | | | | | | | | | | | | |
|  | L | shoot | 88 | ± | 18 | -7.91 | ± | 2.26 | 0.30 | ± | 0.20 | 0.595 | 0.026 | 22.2 | 191 |
|  |  | root | 84 | ± | 18 | -5.37 | ± | 1.84 | 0.34 | ± | 0.19 | 0.557 | 0.033 | 23.8 | 234 |
|  |  | nodule | 91 | ± | 16 | -8.02 | ± | 2.26 | 0.30 | ± | 0.22 | 0.659 | 0.016 | 19.4 | 240 |
|  |  |  |  |  |  |  |  |  |  |  |  |  |  |  |  |
| EDTA-Cu | D | shoot | 85 | ± | 8.2 | -0.03 | ± | 0.006 |  |  |  | 0.758 | 0.007 | 15.1 | 1350 |
|  |  | root | 85 | ± | 5.9 | -0.03 | ± | 0.004 |  |  |  | 0.872 | 0.001 | 10.7 | 1310 |
|  |  | nodule | 77 | ± | 6.0 | -0.02 | ± | 0.004 |  |  |  | 0.842 | 0.002 | 11.0 | 1120 |
|  |  | | | | | | | | | | | | | | |
|  | L | shoot | 89 | ± | 18 | -8.58 | ± | 2.66 | 0.31 | ± | 0.22 | 0.610 | 0.023 | 21.4 | 130 |
|  |  | root | 86 | ± | 18 | -5.87 | ± | 1.93 | 0.36 | ± | 0.21 | 0.579 | 0.029 | 23.2 | 155 |
|  |  | nodule | 92 | ± | 16 | -8.71 | ± | 2.36 | 0.31 | ± | 0.24 | 0.678 | 0.014 | 18.9 | 158 |
|  |  |  |  |  |  |  |  |  |  |  |  |  |  |  |  |
|  | D | shoot | 87 | ± | 9.7 | -72.0 | ± | 18.6 |  |  |  | 0.698 | 0.012 | 16.8 | 0.52 |
| CaCl_2_- Cu |  | root | 88 | ± | 7.9 | -73.9 | ± | 15.3 |  |  |  | 0.788 | 0.005 | 13.8 | 0.51 |
|  |  | nodule | 79 | ± | 7.7 | -67.6 | ± | 14.8 |  |  |  | 0.767 | 0.006 | 13.4 | 0.43 |
|  |  |  |  |  |  |  |  |  |  |  |  |  |  |  |  |
|  | L | shoot |  |  |  | 16.5 | ± | 2.35 | -0.85 | ± | 0.08 | 0.872 | < 0.001 | 18.5 | 0.27 |
|  |  | root |  |  |  | 20.0 | ± | 3.11 | -0.75 | ± | 0.08 | 0.853 | < 0.001 | 20.5 | 0.30 |
|  |  | nodule | 59 | ± | 14 | -1.39 | ± | 0.68 |  |  |  | 0.350 | 0.095 | 26.8 | 6.29 |
| ^a^ SEM - standard error of mean.  ^b^ RMSE - root mean square error.  ^c^ EC_50_ - effective concentration for 50 % decrease in harvest. | | | | | | | | | | | | | | |  |


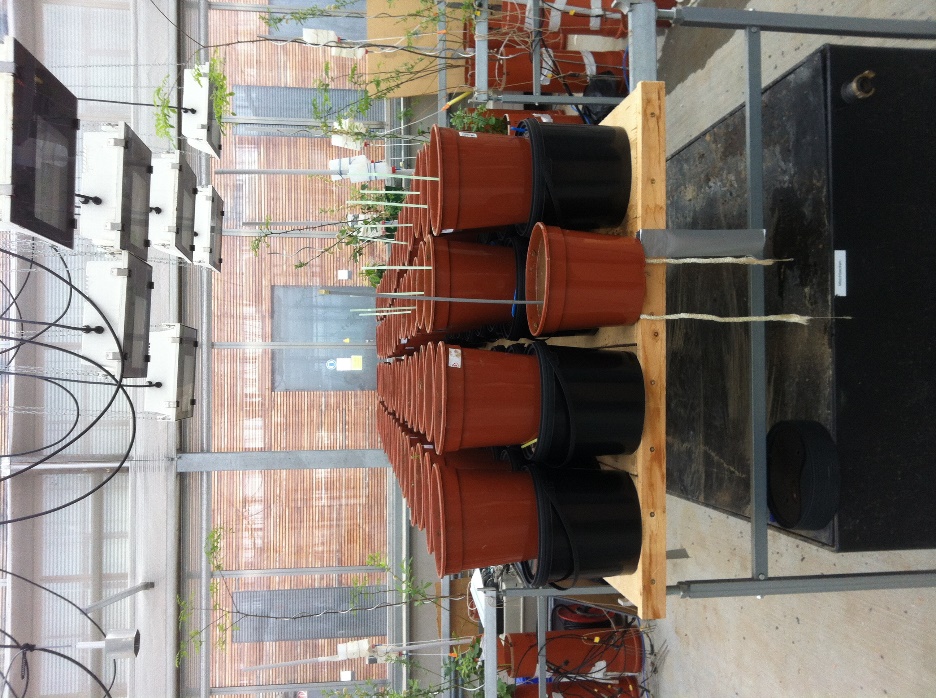

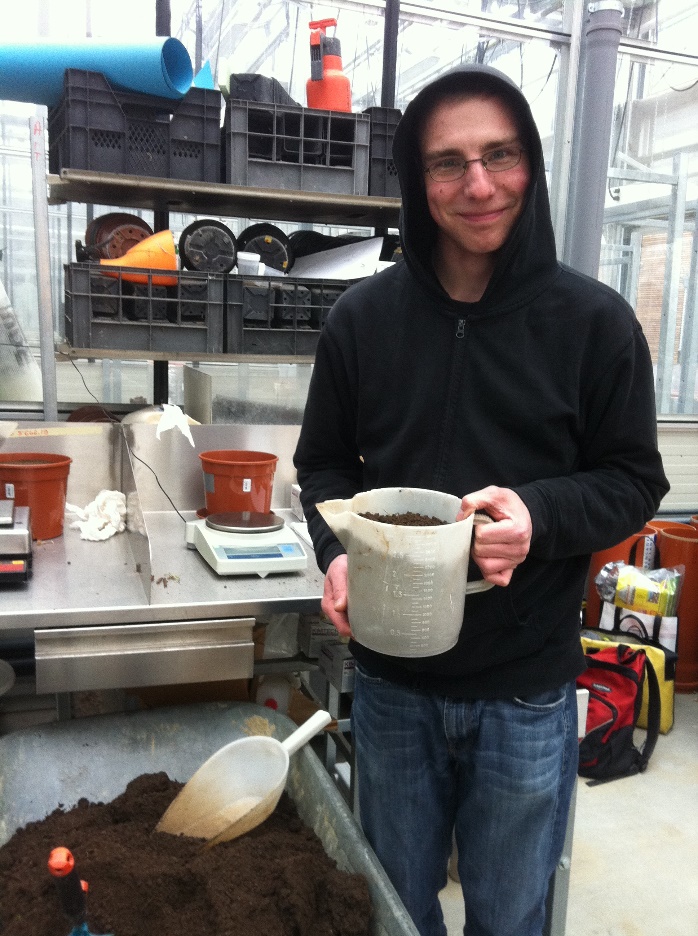


**Supp. Fig. 1** Setup of the experiment with two soils, compacted to 1.2 g soil dry weight cm^-3^ and equipped with glass fiber wicks for passive watering.


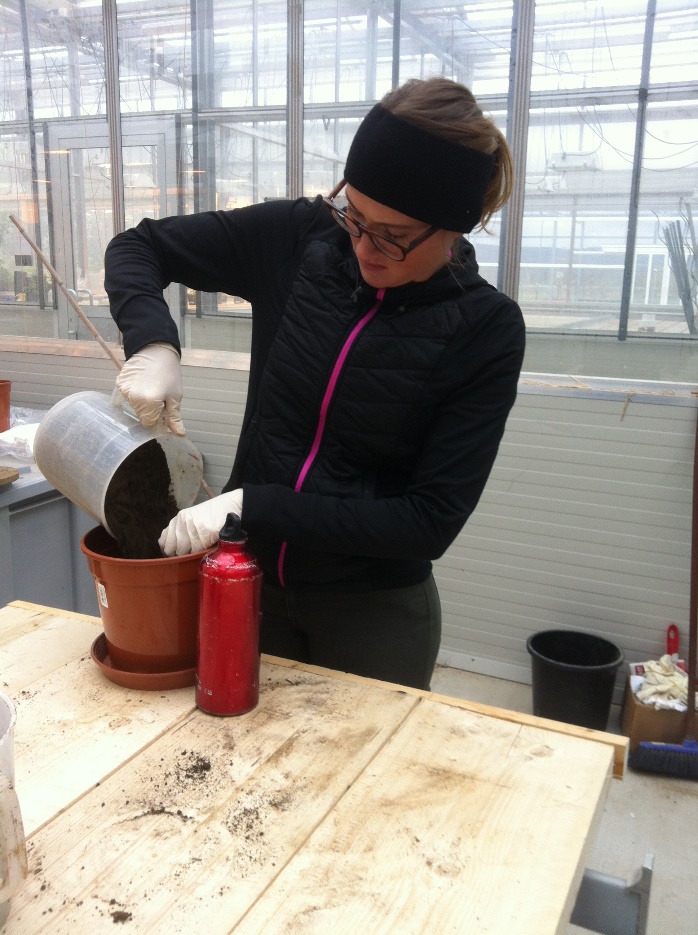

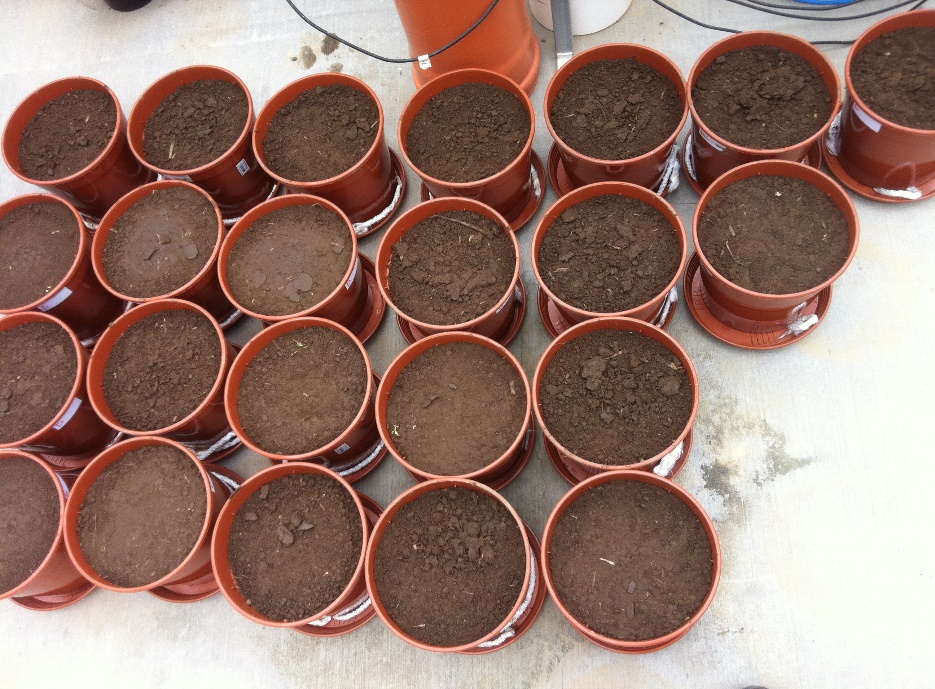

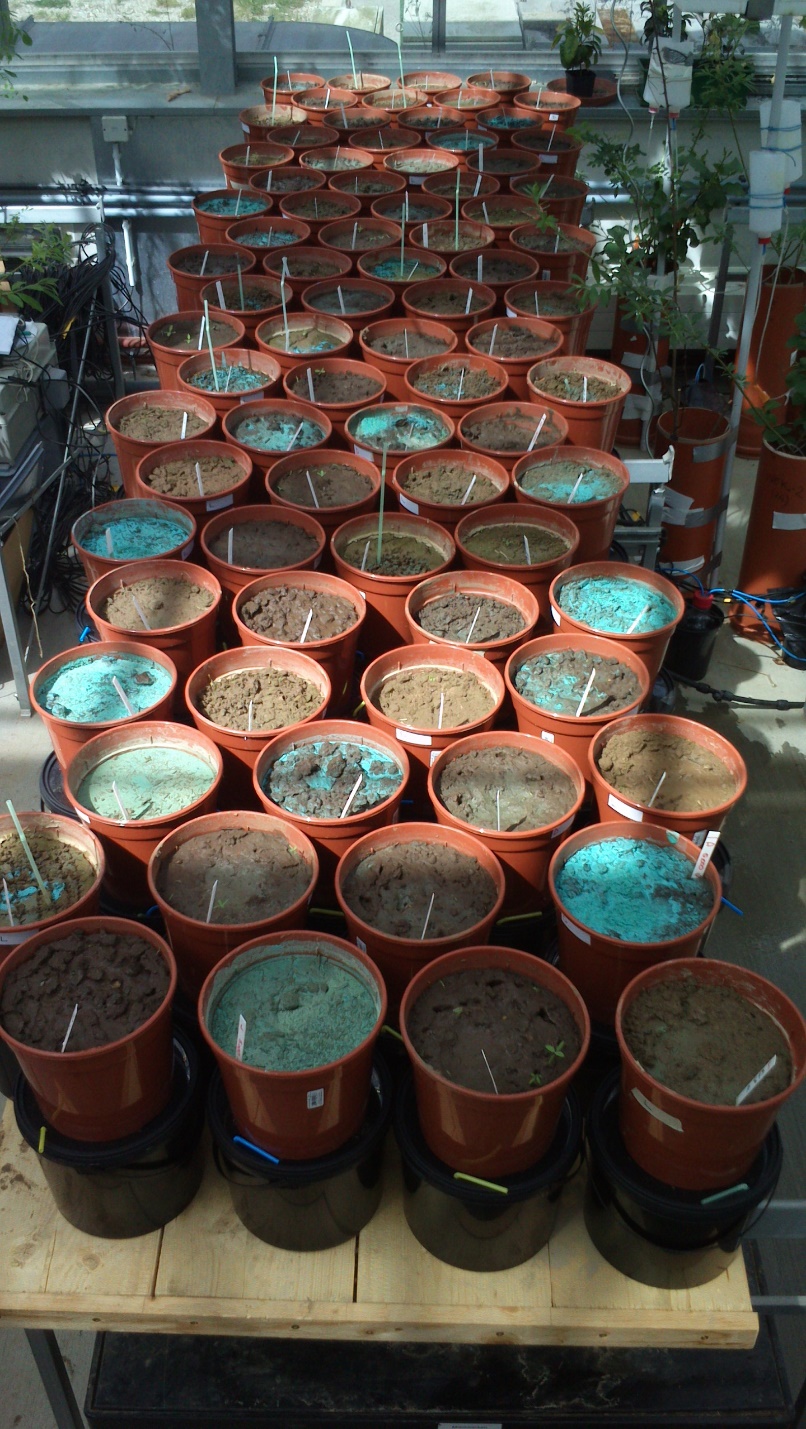

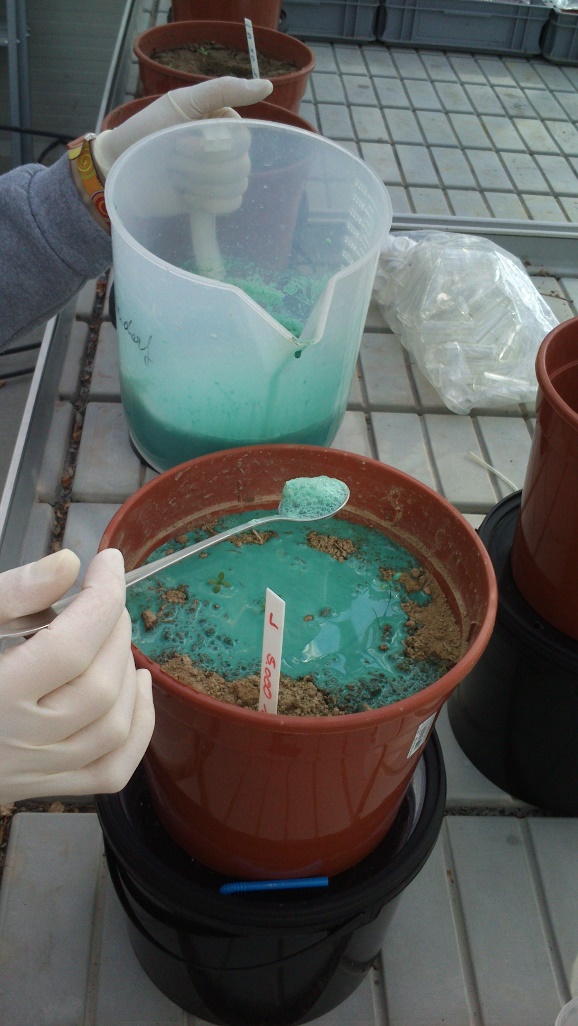

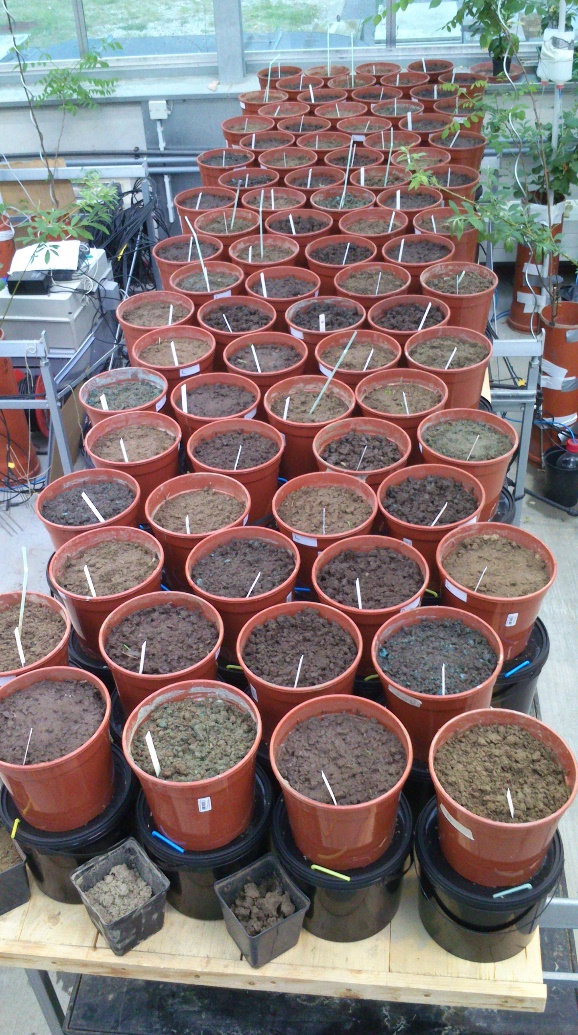


**Supp. Fig. 2** Application of the Cu(OH)_2_-fungicide, two weeks after the second leaching of nitrate (top). One week before sowing the lucerne. Topsoils were scarified and inoculated with various strains of rhizobia (bottom, left).


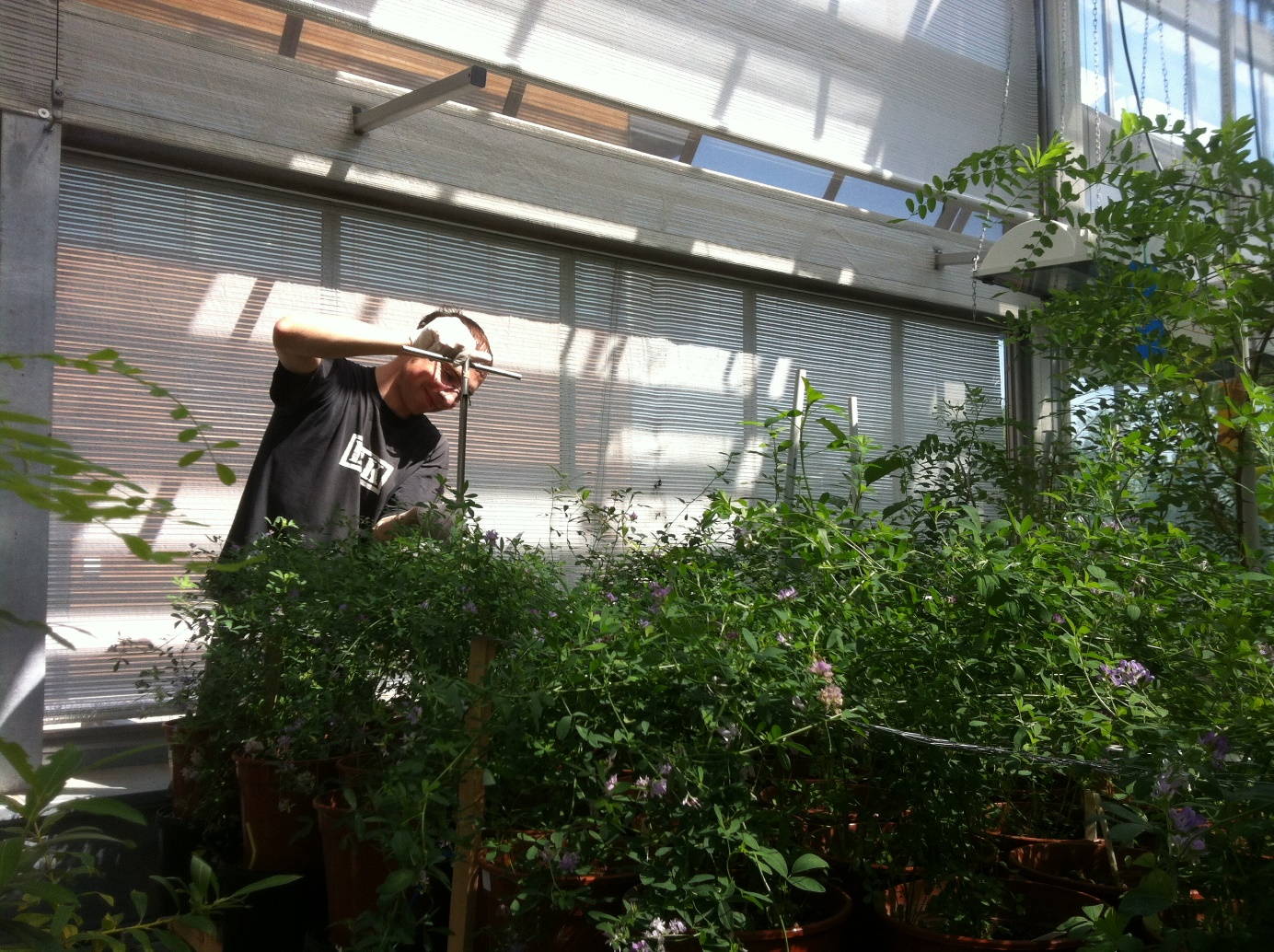


**Supp. Fig. 3** The second soil sampling while plants were still growing on the 92^nd^ day after sowing.


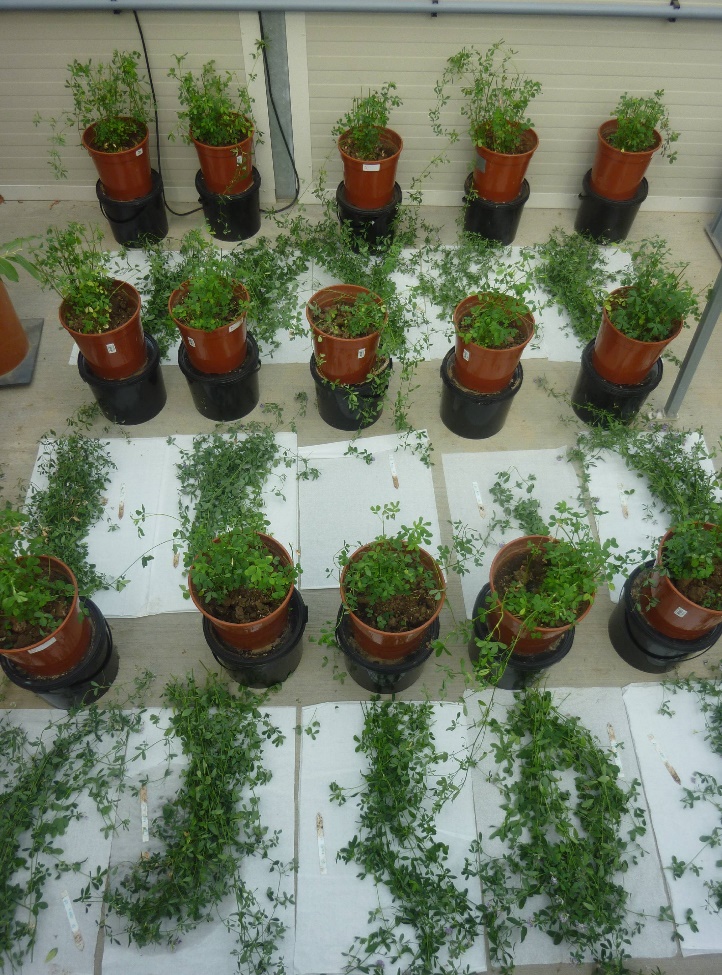

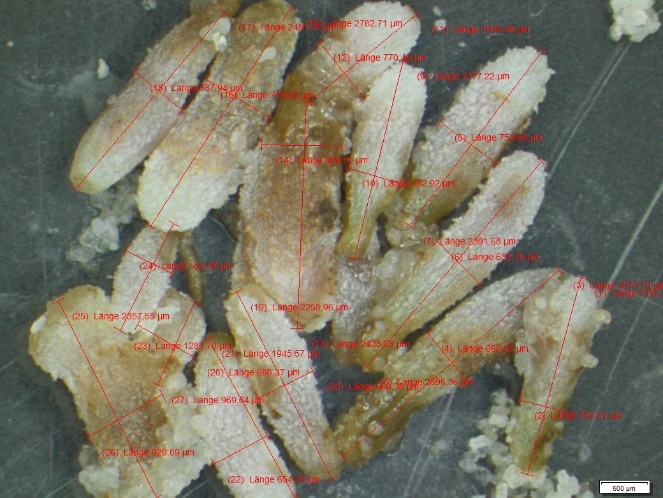

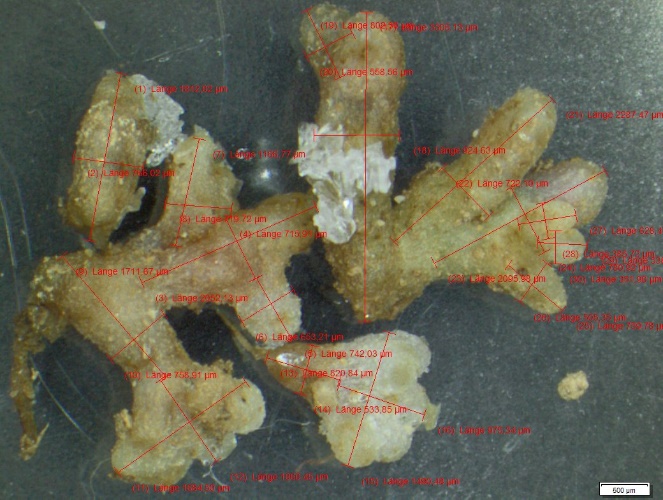


**Supp. Fig. 5** For harvesting the nodules (left) on the 101^st^ and 102^nd^ day after sowing, plants were cut and sampled afterwards, one after another, to maintain 24 hours incubation time for acetylene reduction. The produced ethylene was related to the number and volume of the nodules, respectively. The latter was measured with a microscope (right).


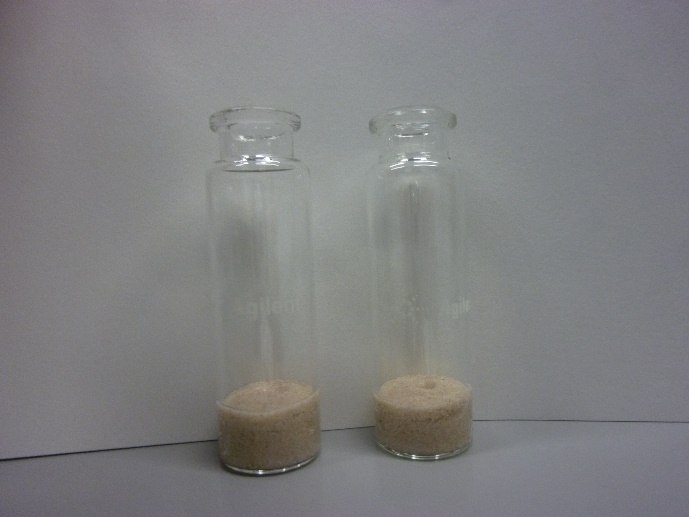

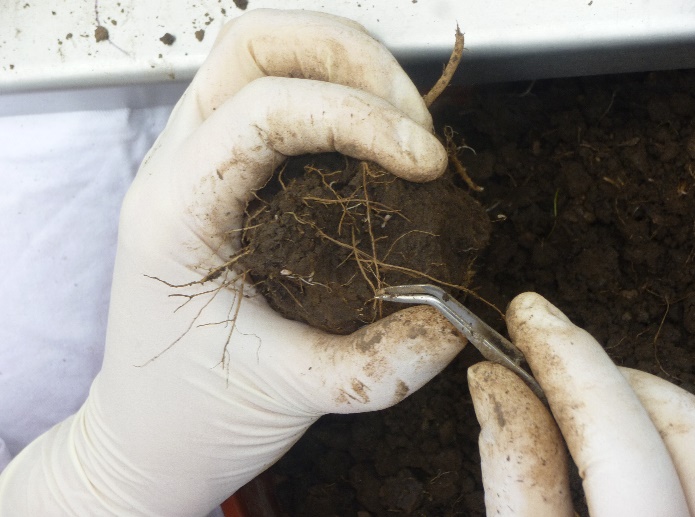


**Supp. Fig. 4** Plants were harvested one week after the second soil sampling, on the 101^st^ and 102^nd^ day after sowing. Ten nodules each were picked out for the acetylene reduction assay (right). GC headspace vials (left) for the acetylene reduction assay contained sea sand and isotonic Ringer solution.


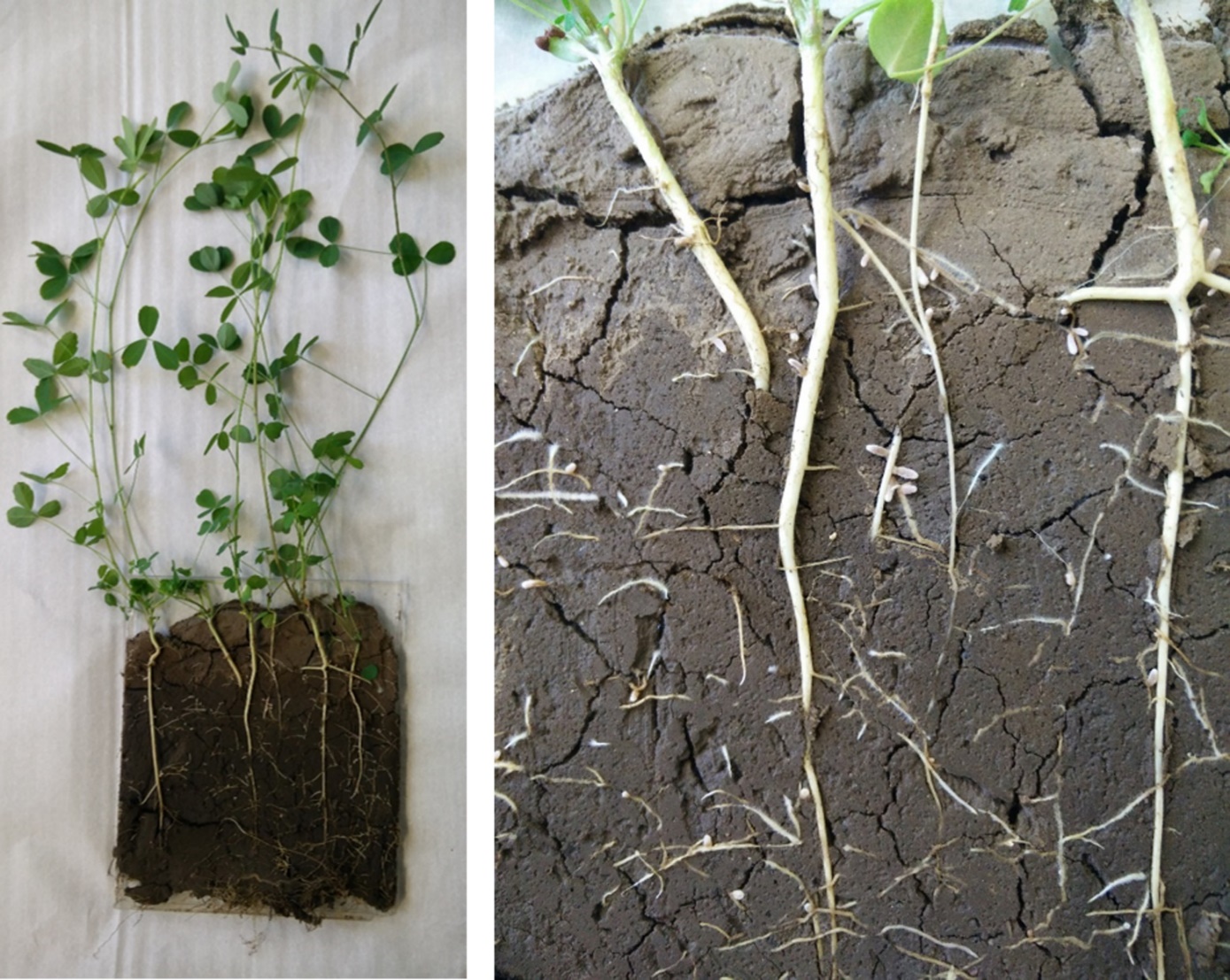


Supp. Fig. 6 Alfalfa (left) and its root nodules (right) after growing ten weeks in rhizotrons on a silt loam contaminated with 0.5 g Cu kg^-1^.


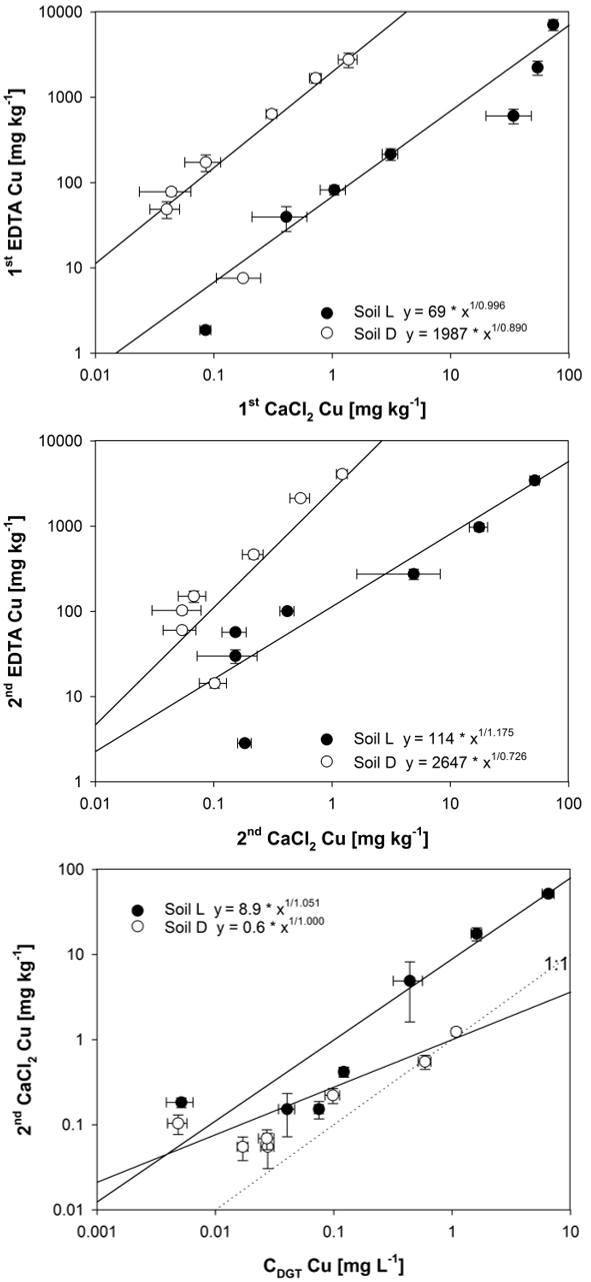


Supp. Fig. 7 Relationship of 0.01 M CaCl_2_-extractable Cu and 0.05 M EDTA-extractable Cu on the 14^th^ (top) and on the 92^nd^ (middle) day after sowing and with Cu in diffusive gradients in thin films (DGT) on the 92^nd^ day after sowing (bottom), according to Freundlich isotherms. The error bars represent standard errors (n=5).


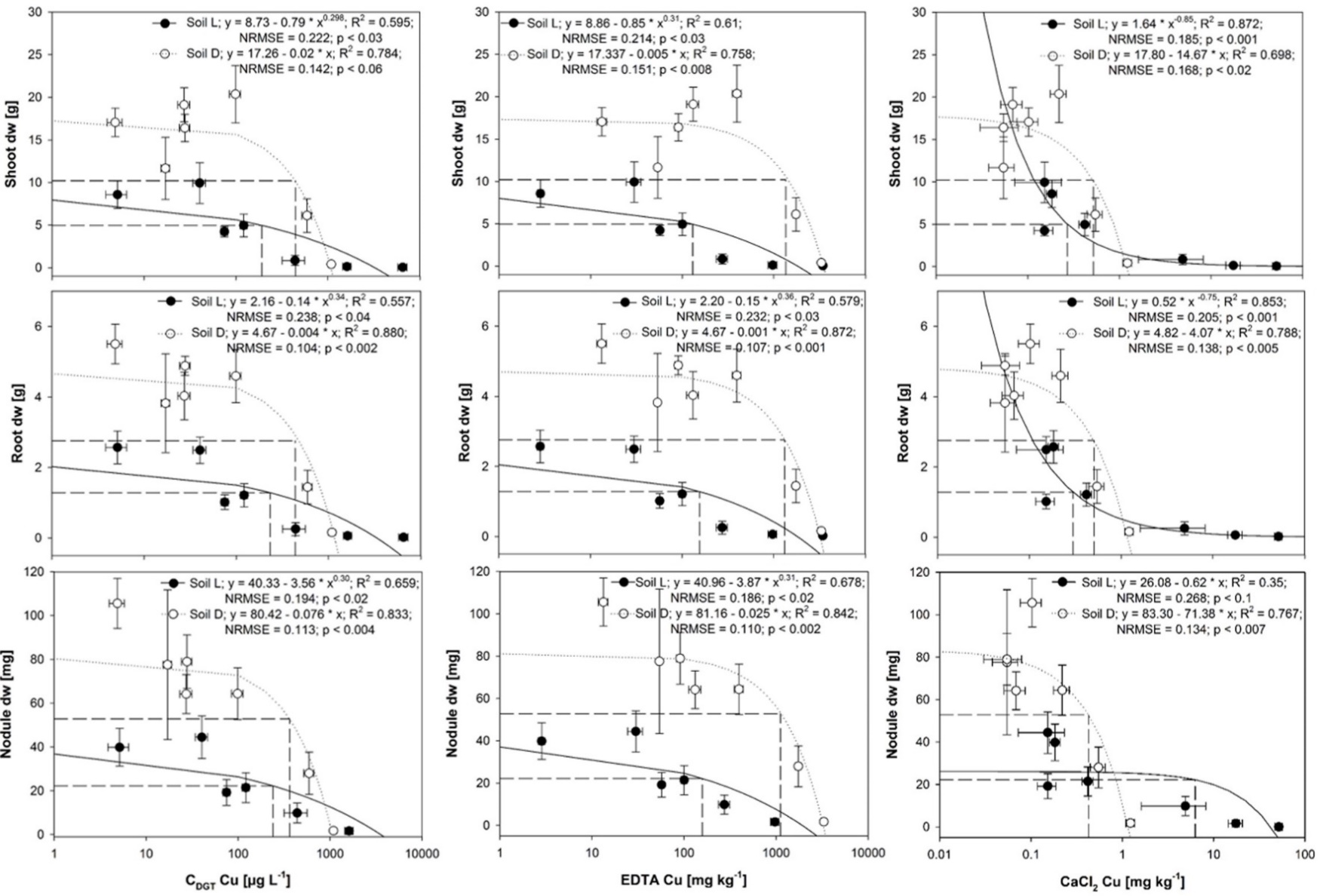


**Supp. Fig. 8** Biomass dry weights (dw) per pot of shoot (top), root (middle) and root nodules (bottom) related to C_DGT_-Cu (left), 0.05 M EDTA-extractable Cu (middle) and 0.01 M CaCl_2_-extractable Cu (right) at the 92^nd^ day after sowing. Dashed lines correspond to soil-specific EC_50_ concentrations. The root mean square error is normalized (NRMSE) on the value range for comparing the regression quality.


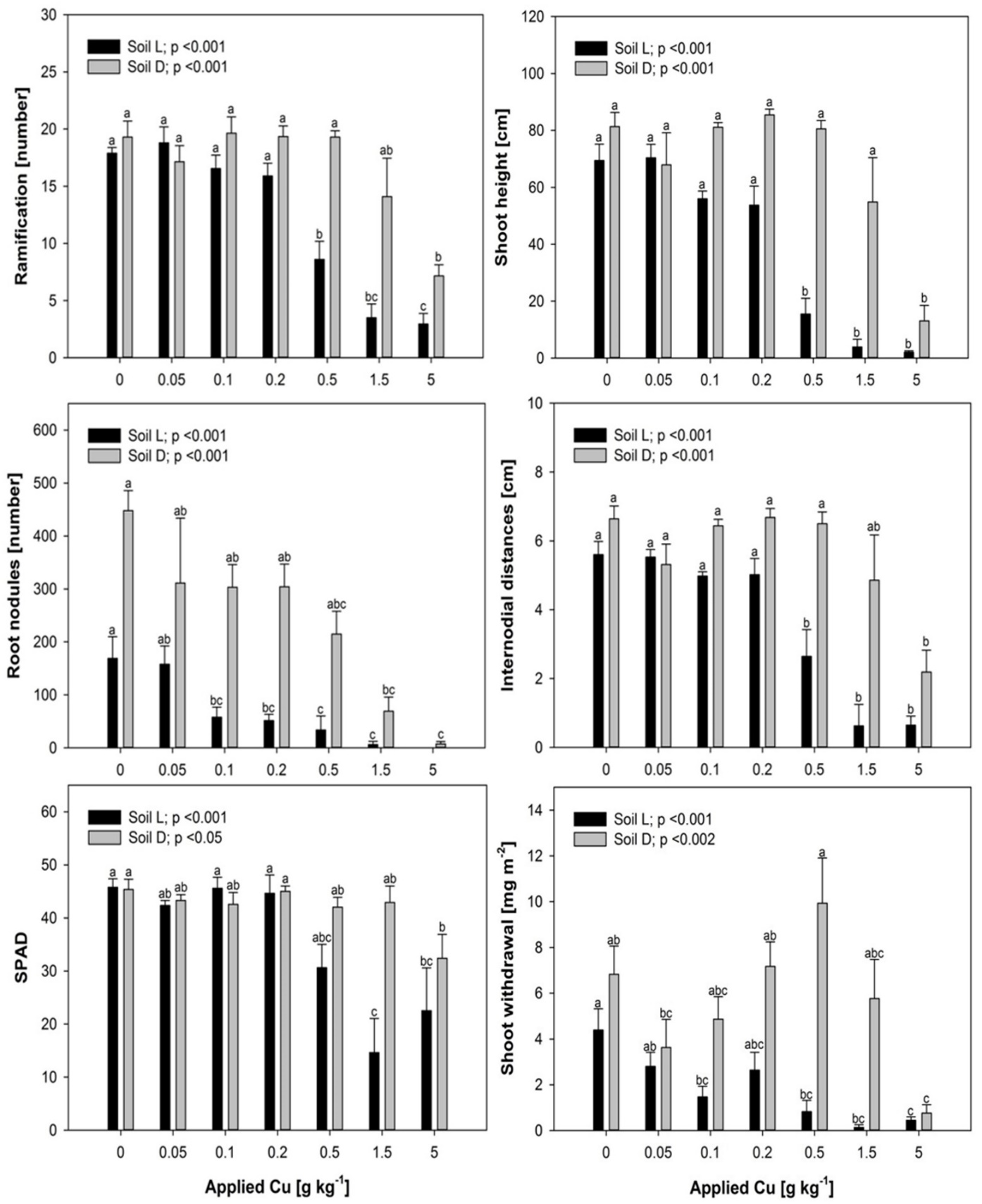


Supp. Fig. 9 Growth factors measured between the 81^st^ and the 84^th^ day after sowing for four plants in each pot, (except for the root nodule number and the shoot withdrawal). Different letters above standard errors (n=5) indicate significant differences between treatments within the same soil.


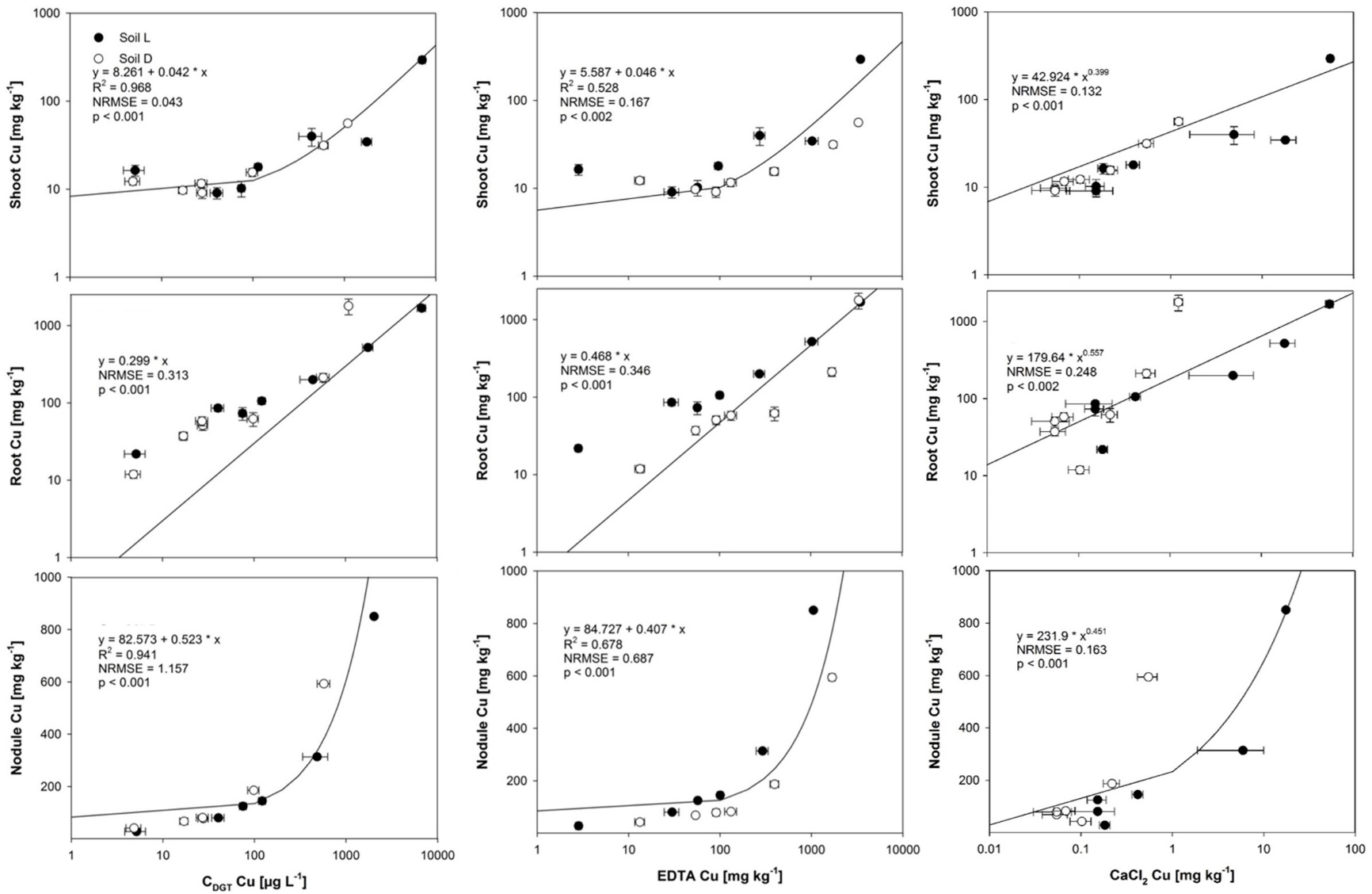


**Supp. Fig. 10** Tissue Cu concentrations in shoot (top), root (middle) and nodule (bottom) related to C_DGT_-Cu (left), 0.05 M EDTA-extractable Cu (middle) and to 0.01 M CaCl_2_-extractable Cu (right) at the 92^nd^ day after sowing. Root Cu concentrations did not differ between soils. Root nodule Cu concentrations are single values of pooled samples within each treatment. The root mean square error is normalized (NRMSE) on the value range for comparing the regression quality.


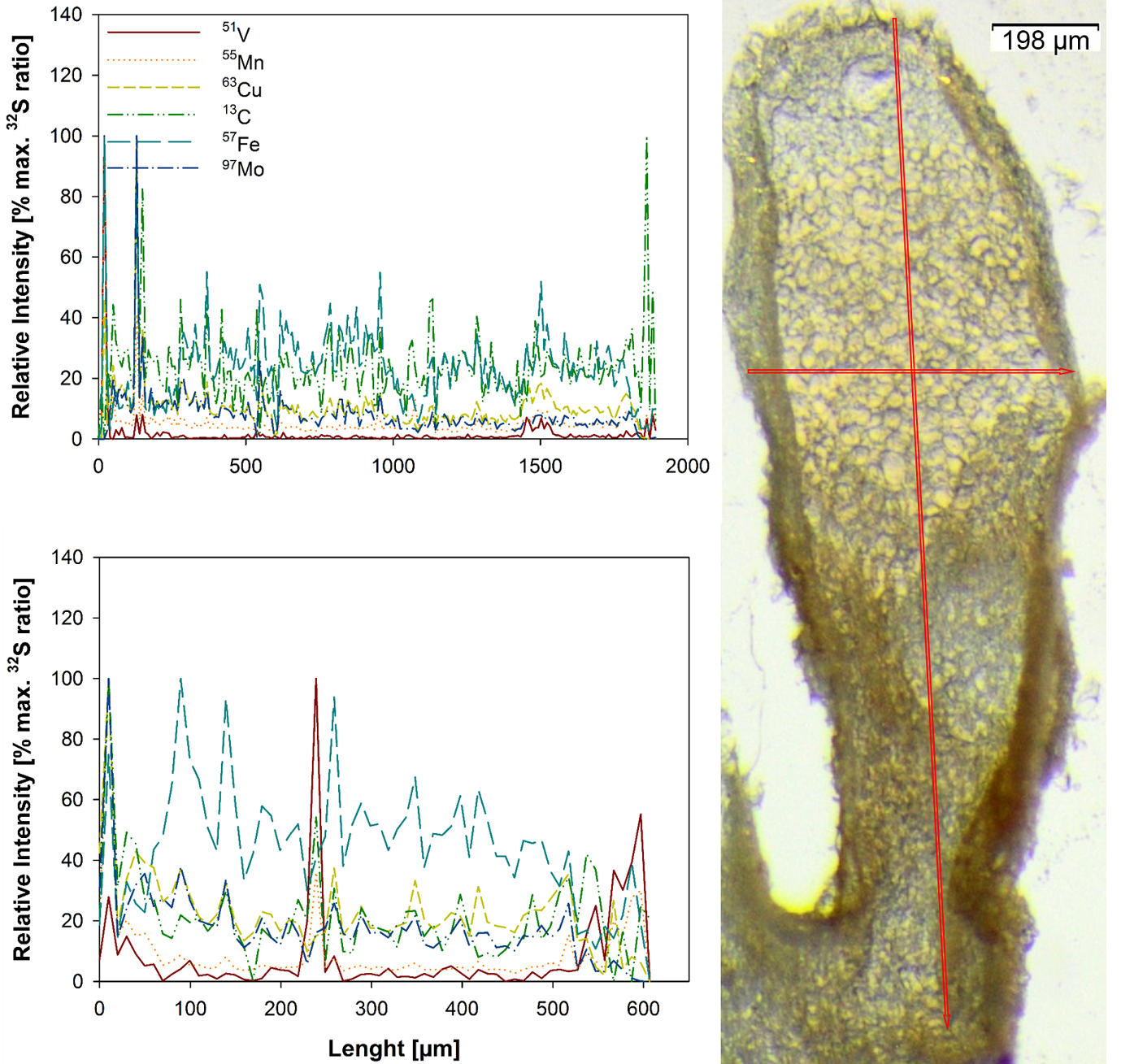
**Supp. Fig. 11** Linescans of a longitudinal (top) and a radial section (bottom) of a *Medicago sativa* root nodule as measured by LA-ICP-MS. Results are scaled on the maximum measured ^32^S-based ratio for reasons of comparability.


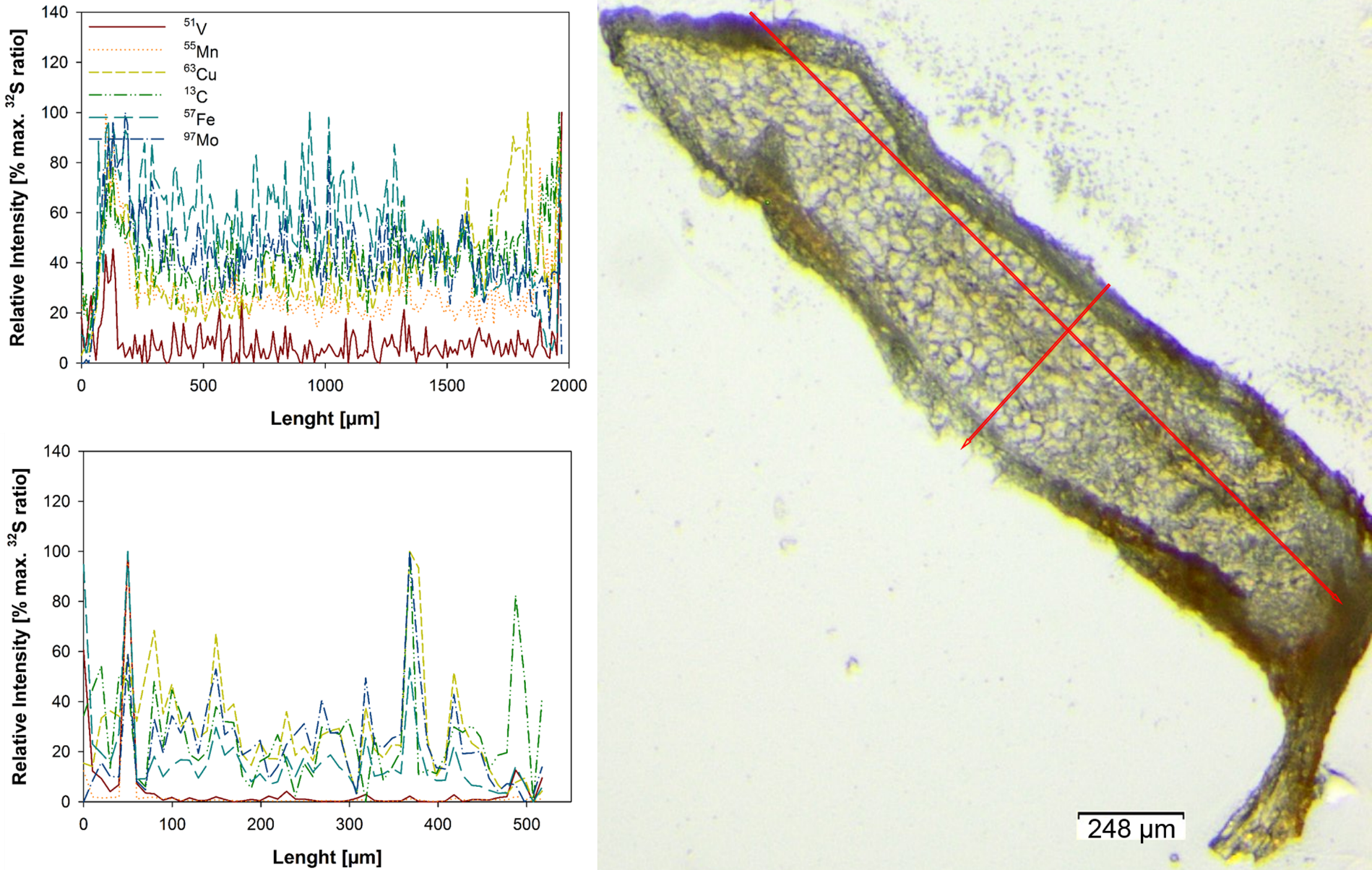


**Supp. Fig. 12** Linescans of a longitudinal (top) and a radial section (bottom) of a *Medicago sativa* root nodule as measured by LA-ICP-MS. Results are scaled on the maximum measured ^32^S-based ratio for reasons of comparability.
